# Production of New Antibacterial 4-Hydroxy-*α*-Pyrones by a Marine Fungus *Aspergillus niger* Cultivated in Solid Medium

**DOI:** 10.3390/md17060344

**Published:** 2019-06-10

**Authors:** Lijian Ding, Lu Ren, Shuang Li, Jingjing Song, Zhiwen Han, Shan He, Shihai Xu

**Affiliations:** 1 Li Dak Sum Yip Yio Chin Kenneth Li Marine Biopharmaceutical Research Center, College of Food and Pharmaceutical Sciences, Ningbo University, Ningbo 315832, China; dinglijian@nbu.edu.cn (L.D.); renlurenlu@163.com (L.R.); lishuang9892@163.com (S.L.); 13123838771@163.com (J.S.); hanzhiwen1999@163.com (Z.H.); 2 Department of Chemistry, College of Chemistry and Materials Science, Jinan University, Guangzhou 510632, China

**Keywords:** pyrone, sponge, marine fungi, ECD, antibacterial

## Abstract

Four 4-hydroxy-*α*-pyrones including three new ones named nipyrones A–C (**1**–**3**) together with one known analogue germicidin C (**4**) were discovered from a marine sponge-derived fungus *Aspergillus niger* cultivated in a solid rice culture. Their structures and absolute configurations were elucidated through a combination of spectroscopic data and electronic circular dichroism (ECD) calculations as well as comparison with literature data. Compounds **1**–**4** were evaluated for their antibacterial activities against five pathogenic bacteria (*Staphylococcus aureus*, *Escherichia coli*, *Bacillus subtilis*, methicillin-resistant *Staphylococcus aureus*, and *Mycobacterium tuberculosis*). Compound **3** showed promising activity against *S. aureus* and *B. subtilis*, with minimum inhibitory concentration (MIC) values of 8 μg/mL and 16 μg/mL, respectively, and displayed weak antitubercular activities against *M. tuberculosis*, with MIC value of 64 μg/mL, while compounds **1** and **2** exhibited moderate antibacterial efficacy against four pathogenic bacteria with MIC values of 32–64 μg/mL.

## 1. Introduction

In recent years, sponge-derived fungi have represented a potential resource for discovery of novel bioactive molecules [1,2]. Numerous secondary metabolites with a broad spectrum of bioactivities have been isolated from sponge-derived fungi, inclusive of alkaloids [3], terpenoid [4], polyketides [5], and peptides [6]. α-Pyrones are one of polyketide-biosynthetic skeletons, characterized by six-membered unsaturated cyclic scaffold containing a lactone [7]. α-Pyrones can be widely found in the fungi and actinomycetes [8,9,10], and these molecules demonstrated a wide range of extraordinary biological activities, such as antimicrobial [11], anti-inflammatory [12], cytotoxic [13], and quorum sensing signaling molecules [14,15]. 

**Figure 1:**
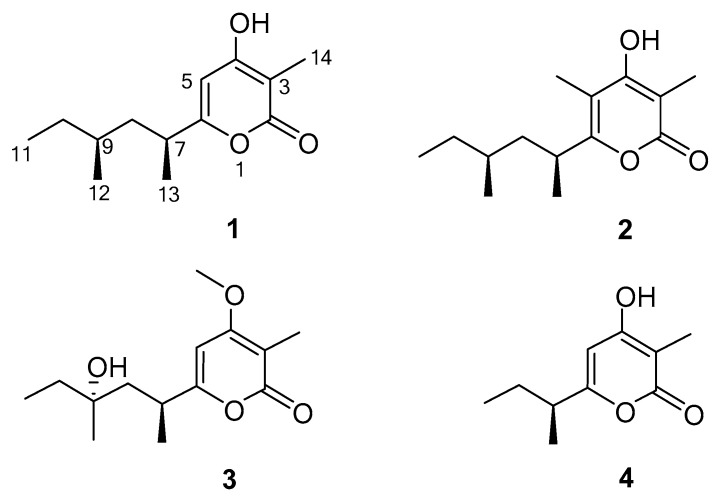
Structures of compounds **1**–**4**.

Members of the genus *Aspergillus* are well known to produce chemically diverse secondary metabolites, many of which have been developed to therapeutic leads for human health [16,17,18,19]. During our ongoing search for sponge-derived fungi capable of producing antibiotics, a sponge-derived fungus *Aspergillus niger* LS24 showed antimicrobial activities. HPLC-UV profile of crude extract of *A. niger* LS24 grown on solid rice medium indicated the presence of various α-pyrone derivatives with the characteristic UV absorption similar to that of germicidin C [20]. Antibacterial-guided fractionation of the EtOAc extract from a scale-up culture led to the isolation and identification of three new 4-hydroxy-α-pyrones, named nipyrones A–C (**1**–**3**) and one known analogue, germicidin C (**4**) (Figure 1). Compounds **1**–**4** were evaluated for their antibacterial activities against five pathogenic bacteria. Herein, the isolation, structure elucidation, and antibacterial evaluation of these α-pyrones are described.

## 2. Results and Discussion

### 2.1. Structure Elucidation

**Figure 2:**
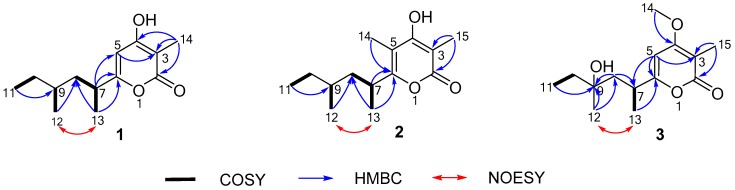
Key HMBC, COSY, and NOESY correlations of **1**–**3**.

**Figure 3:**
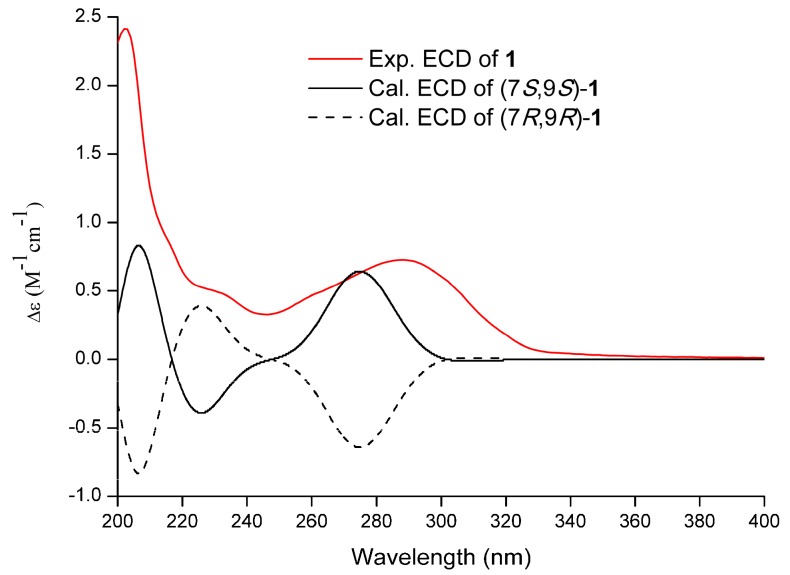
Experimental and calculated electronic circular dichroism (ECD) spectra of **1** in methanol.

Nipyrone A (**1**) was obtained as a colorless oil. The molecular formula of **1** was assigned as C_13_H_20_O_3_ by HRESIMS data and gave an [M + H]^+^ peak at *m/z* 225.1487, suggesting four degrees of unsaturation. Its UV absorption at 290 nm indicated the presence of a conjugated α-pyrone chromophore [21] (Figure S2). The ^1^H-NMR spectrum (Table 1) of **1** displayed one olefinic proton at δ_H_ 6.16 (s, H-5), two methylenes at δ_H_ 1.70 (m, H-8), 1.19 (m, H-8), 1.27 (m, H-10), and 1.10 (m, H-10), two methines at δ_H_ 2.64 (m, H-7) and 1.27 (m, H-9), two methyl doublets at δ_H_ 0.84 (d, *J* = 6.4 Hz, H_3_-12) and 1.19 (d, *J* = 6.9 Hz, H_3_-13), one methyl triplet at δ_H_ 0.82 (t, *J* = 7.3 Hz, H_3_-11), and one methyl singlet at δ_H_ 1.97 (s, H_3_-14). Analysis of ^13^C NMR and DEPT spectra of **1** classified the 14 carbons into four methyls, two methylenes, three methines, and four nonprotonated carbons. HMBC correlations (Figure 2) of H_3_-14/C-2 (δ_C_ 167.3), C-3 (δ_C_ 98.7), and C-4 (δ_C_ 168.7) as well as H-5/C-3 (δ_C_ 98.7) in the HMBC spectrum suggested the existence of the 4-hydroxy-3-methyl *α*-pyrone skeleton (Figure 2 and Figure S7). The structure of a 1,3-dimethylpentyl group was further verified by the COSY correlations (H-7/H_2_-8/H_3_-13, H-9/H_3_-12, and H-10/H_3_-11) and HMBC correlations from H_3_-12/H_3_-13 to C-8 (δ_C_ 41.7) and H_3_-11 to C-9 (δ_C_ 32.1). The 1,3-dimethylpentyl group was connected to C-6 (δ_C_ 167.3) of 4-hydroxy-3-methyl α-pyrone moiety, supported by the HMBC correlations of H-7/C-5 (δ_C_ 100.0) and C-6 together with H_3_-13/C-6 (Figure 2). Thus, the planar structure of **1** was established as 4-hydroxy-3-methyl-6-(-4-methylhexan-2-yl)-2*H*-pyran-2-one. The relative configuration of **1** was established by the NOESY data (Figure S9). The NOE correlation between H_3_-12/H_3_-13 clarified H_3_-12 and H_3_-13 to be cofacial of the side chain (Figure 2). Thus, the absolute configuration at C-7 and C-9 of **1** was identified as 7*S*,9*S* or 7*R*,9*R*. The absolute configuration of **1** was further determined by comparing the experimental electronic circular dichroism (ECD) spectrum of **1** with the correspondingly time-dependent density functional theory (TDDFT)-calculated one. The Cotton effects of the experimental ECD spectrum of **1** matched very well with the calculated ECD spectrum for the model molecule of 7*S*,9*S* at the B3LYP/6-311 + G(d, p) level (Figure 3 and see Supplementary Materials), thus confirming its absolute configuration.

Nipyrone B (**2**) was also obtained as a colorless oil with a molecular formula of C_14_H_22_O_3_ as determined by HRESIMS data with one CH_2_ more than **1**. The spectroscopic data of **2** (Table 1) were highly identical to that of **1** except for the absence of one olefinic proton and an additional methyl singlet. The additional methyl group was located at C-5 (δ_C_ 106.8, Table 1) by HMBC correlation from H_3_-14 to C-6 (Figure 2 and see Supplementary Materials). The absolute configuration of **2** tentatively led to deduction of the same as **1** based on the biosynthetic consideration and specific rotation.

Nipyrone C (**3**) was isolated a as a colorless oil, and its molecular formula was assigned as C_14_H_23_O_4_ based on the HRESIMS ([M + H]^+^ *m*/*z* 255.1591) and ^13^C NMR data. The 1D and 2D NMR spectroscopic data for **3** (Table 1) closely resembled those of **1**. Significant differences in the NMR data for **3** were only found in the resonances assigned to an additional hydroxy group and an additional methoxy group. Moreover, the C-9 position in **1** replaced by a hydroxy group in **3** was confirmed by chemical shift C-9 (δ_C_ 72.9). The attachment of the methoxy group (C-14, δ_C_ 56.1) at C-4 was supported by the HMBC correlation of H_3_-14/C-4 (see Supplementary Materials). Based on the biosynthetic point of view and specific rotation, the absolute configuration of **3** might be assigned to be 7S,9*R*. 

**Scheme 1:**
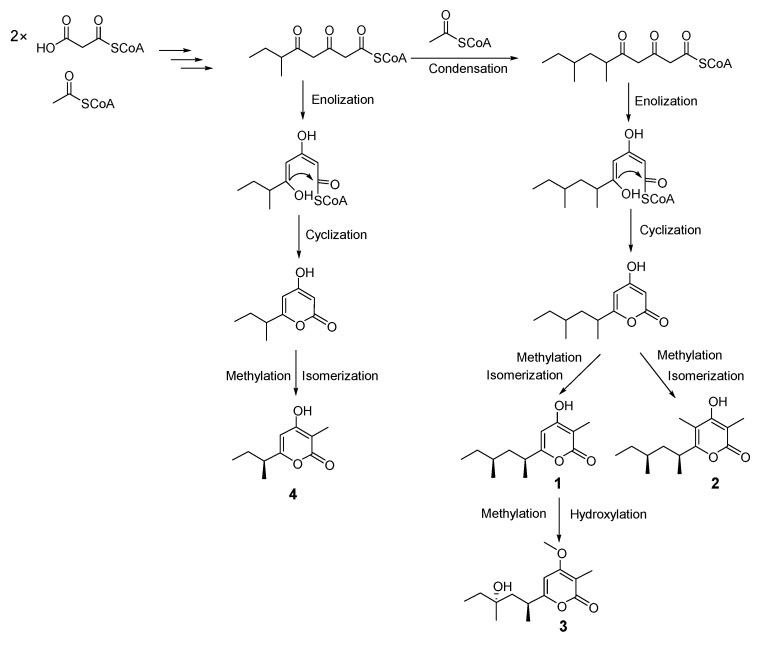
Proposed biosynthetic pathway of **1**–**4**.

In addition, compound **4** was identified as germicidin C by comparison of its spectral data with those reported in [20]. Many α-pyrone-based secondary metabolites biosynthesized by polyketide synthetase (PKS) pathway have been widely reported, uncovering their biosynthetic gene clusters [7,22,23]. We proposed that the polyketide chain primed with acetyl-CoA and malonyl-CoA was elongated, enolized, cyclized, methylated, hydroxylated, and released as the corresponding four 4-hydroxy-α-pyrones. A probable biosynthesis pathway of **1**–**4** is illustrated in Scheme 1.

### 2.2. Biological Activities

The antibacterial activities of **1**–**4** were evaluated against four bacteria, including Gram-positive and Gram-negative bacteria, using broth micro-dilution method within a concentration range of 256–1 µg/mL. Four pathogenic bacteria, including *S. aureus*, *E. coli*, *B. subtilis*, and methicillin-resistant *S. aureus* (MRSA) were performed. The results are shown in Table 2. Compound **3** exhibited significant inhibitory activity against *S. aureus* and *B. subtilis*, with minimum inhibitory concentration (MIC) values of 8 and 16 µg/mL, respectively. Compounds **1**, **2**, and **4** exhibited moderate antimicrobial effect against *S. aureus*, *E. coli*, and *B. subtilis*, with MIC values in range of 32–64 µg/mL. Compounds **1**–**4** displayed weak antibiotic capacity against MRSA. Compound **3** possessed weak antitubercular activities against *M. tuberculosis* (MIC, 64 µg/mL).

## 3. Materials and Methods 

### 3.1. General Experimental Procedures

Optical rotations were determined with a P-2000 digital polarimeter (JASCO, Hachioji, Japan). UV spectra were obtained with a NADE Evolution 201 spectrophotometer (ThermoFisher, Waltham, MA, USA). IR spectra were measured on a Nicolet iS5 spectrometer (ThermoFisher, Waltham, MA, USA). NMR data were carried out at ambient temperature on a Varian 600 MHz (Palo Alto, CA, USA) spectrometer operating at 600 (^1^H) and 150 (^13^C) MHz. HRESIMS data were recorded on an Agilent Technologies 6520 Accurate Mass Q-TOF LC/MS spectrometer equipped with an ESI source (Agilent Technologies, Santa Clara, CA, USA). Medium-pressure liquid chromatography (MPLC) was performed on a FLEXA Purification System (Bonna-Agela Technologies Co., Tianjin, China) using a 15 µm ODS column (Santai Technologies, Inc., Changzhou, China). Semi-preparative HPLC was performed on an YMC-Pack Pro C18 RS column (5 μm, 250 × 10 mm id; YMC, Kyoto, Japan) with a Waters 600 separation system coupled with a Waters 2998 Photodiode Array detector (Waters, MA, USA). Column chromatography (CC) was performed on silica gel (200–300 mesh, Qingdao Haiyang Chemical Factory, Qingdao, China) and Sephadex LH-20 (25–100 µm; Pharmacia, Uppsala, Sweden).

### 3.2. Fungal Material

Marine sponge *Haliclona* sp. was collected at Lingshui, Hainan Province, China. The sponge tissue was cut into small pieces of about 0.1 cm^3^ each, which were homogenized with sterile seawater. 20 μL of the diluted homogenate (1:100, sterile seawater) was inoculated in PDA agar plates, which were incubated for periods of 3 days to 4 weeks for purifying fungal colonies. 15 fungal isolates were obtained. Among them, the EtOAc extract of the fungal strain LS24 showed antimicrobial activities. Surprisingly, its extract showed stronger antimicrobial effects (MICs, 32–128 μg/mL against different pathogenic bacteria) when grown on solid rice medium in comparison to liquid medium (MICs, >128 μg/mL against different pathogenic bacteria). The fungal strain LS24 was identified using morphological studies, DNA amplification, and the internal transcribed spacer region (ITS) sequencing (GenBank accession ID: KX290301, 100% similarity). The isolate was stored on PDA medium (potato 200 g, dextrose 20 g, sea salt 35 g and agar 15 g in 1.0 L of H_2_O, pH 7.4–7.8) slants at 4 °C. A voucher strain was preserved at College of Food and Pharmaceutical Sciences, Ningbo University, Ningbo, China.

### 3.3. Fermentation

The fungus *A. niger* was cultured on PDA agar plate at 28 °C for 7 days. The fungal colony was further inoculated into the PDB medium (potato 200 g, dextrose 20 g, and sea salt 35 g in 1.0 L of H_2_O, pH 7.4–7.8) at 28 °C for 3 days on a rotating shaker (180 rpm). Then, a large-scale fermentation of the strain was performed. The fungal seed broth (20 mL) was added to 10 flasks (1000 mL), each containing 100 g rice and 160 mL water. These flasks were incubated at 28 °C for 30 days under static conditions.

### 3.4. Extraction and Isolation

All the fermented materials were extracted with 5 L EtOAc three times to afford a brown extract (8 g). The EtOAc extract was subjected to vacuum liquid chromatography (VLC) on a silica gel column (6 × 15 cm, 200–300 mesh) using step gradient elution with petroleum ether/EtOAc (from 20:1 to 0:1, *v*/*v*) to obtain seven fractions (Fr.1–7) according to HPLC analysis. Fraction 4 was further chromatographed over a Sephadex LH-20 column, eluted with CH_3_OH and CH_2_Cl_2_ (1:1, *v*/*v*), yielding three subfractions (Fr.4.A–C). Fr.4.B (300 mg) was further separated into six subfractions (Fr.4.B.1–6) by ODS silica gel MPLC eluting with MeOH/H_2_O (30–100%, 120 min, flow rate 20 mL/min) to obtain nine subfractions (Fr.4.B.1–9). Fr.4.B.3 was separated by semipreparative HPLC (35% MeCN/H_2_O, 2 mL/min, detected at 290 nm) to provide **1** (1.3 mg, *t*_R_ 32 min) and **2** (1.2 mg, *t*_R_ 34 min). Meanwhile, Fr.4.B.7 was purified by semipreparative HPLC (40% MeCN/H_2_O, 2 mL/min, detected at 290 nm) to yield **3** (1.1 mg, *t*_R_ 30 min) and **4** (3.1 mg, *t*_R_ 32 min).

Nipyrone A (**1**): colorless oil; ${\left[\alpha \right]}_{D}^{\mathrm{25}}$ +43 (*c* 0.23, MeOH); CD *λ*_max_ (Δε) 203(+2.41), 288 (+0.72) nm; UV (MeOH) (log ε) *λ*_max_ 290 (4.19) nm; IR (KBr) *ν*_max_ 3093, 2964, 2930, 2876, 2680, 1662, 1582, 1459, 1415, 1375, 1245, 1152, 1126, 1057, 972, 932, 876, 837, 760, 705 cm^−1^; ^1^H and ^13^C NMR data, see Table 1; HRESIMS *m/z* 225.1487 [M + H]^+^ (calcd for C_13_H_21_O_3_, 225.1485).

Nipyrone B (**2**): colorless oil; ${\left[\alpha \right]}_{D}^{\mathrm{25}}$ +72 (*c* 1.13, MeOH); UV (MeOH) (log ε) *λ*_max_ 286 (3.86) nm; IR (KBr) *ν*_max_ 3219, 2963, 2930, 2875, 1705, 1671, 1606, 1568, 1459, 1408, 1377, 1232, 1156, 1093, 1035, 958, 873, 806, 761 cm^−1^; ^1^H and ^13^C NMR data, see Table 1; HRESIMS *m/z* 239.1639 [M + H]^+^ (calcd for C_14_H_23_O_3_, 239.1628).

Nipyrone C (**3**): colorless oil; ${\left[\alpha \right]}_{D}^{\mathrm{25}}$ +35 (*c* 0.12, MeOH); UV (MeOH) (log ε) λ_max_ 299 (3.92) nm; IR (KBr) *ν*_max_ 3429, 3101, 2967, 2929, 2876, 1686, 1642, 1566, 1462, 1406, 1380, 1325, 1251, 1192, 1141, 1099, 1032, 968, 941, 905, 804, 756 cm^−1^; ^1^H and ^13^C NMR data, see Table 1; HRESIMS *m/z* 255.1591 [M + H]^+^ (calcd for C_14_H_23_O_4_, 255.1591).

### 3.5. Antibacterial Assay

The antibacterial effects of compounds **1**–**4** were evaluated against Gram-positive bacteria *S. aureus* ATCC 25923, *B. subtilis* ATCC 6633, methicillin-resistant *S. aureus* ATCC 43300 (MRSA), and Gram-negative bacterium *E. coli* ATCC 25922 according to a previously described method [24]. The test compounds were dissolved in DMSO (1 mg/mL for **1**–**4**). The minimum inhibitory concentration (MIC) values were defined as the lowest concentration of test compound that inhibited the growth of more than 99% of the bacterial population after overnight incubation in 96-well microtiter plates, as detected by eye. Briefly, 100 μL of each bacterial solution was inoculated in each well (10^5^ CFU/mL) and added with 100 µL of each compound solution and control in triples. Microplates were incubated for 24 h at 37 °C. The final concentrations of each test compound in the wells were in the range of 256–1 μg/mL using sequential 2-fold serial dilutions. The final DMSO concentration was maintained at 0.5% by adding the medium. Chloramphenicol and DMSO were used as the positive control and the negative control, respectively. The detailed method of the antitubercular activity of compounds **1**–**4** against *M. tuberculosis* H37Rv was described by the agar proportion method based on the previous report [25]. The blank control was DMSO. Ethambutol was used as a positive control.

## 4. Conclusions

In summary, three new 4-hydroxy-α-pyrone derivatives, nipyrones A–C (**1**–**3**) along with one known analogue germicidin C (**4**) were isolated from a marine sponge-derived fungus *A. niger* grown on a solid rice culture. These 4-hydroxy-α-pyrone derivatives have in common the differences in functional group substitution and side chain length. Biogenetically, nipyrones A–C (**1**–**3**) are presumably originated from the polyketide pathway. This study further expanded the structural diversity of naturally occurring α-pyrone derivatives. Notably, compound **3** displayed significant inhibitory effects on two pathogenic bacteria, *S. aureus* and *E. coli* and may be considered to have potential as an antibiotic agent.

## Figures and Tables

**Table 1:** ^1^H (600 MHz) and ^13^C NMR (150 MHz) data for compounds **1**–**3** (CDCl_3_).

No.	1		2		3	
	δ_C_, Type	δ_H_ (*J* in Hz)	δ_C_, Type	δ_H_ (*J* in Hz)	δ_C_, Type	δ_H_ (*J* in Hz)
2	167.3, C	-	166.9, C	-	165.9, C	-
3	98.7, C	-	98.2, C	-	100.8, C	-
4	168.7, C	-	165.7, C	-	166.1, C	-
5	100.0, CH	6.16, s	106.8, C	-	93.2, CH	6.04, s
6	167.3, C	-	162.1, C	-	168.6, C	-
7	36.1, CH	2.64, m	32.3, CH	3.00, m	35.1, CH	2.85, m
8	41.7, CH_2_	1.70, m; 1.19, m	41.5, CH_2_	1.79, m; 1.19, m	45.5, CH_2_	2.06, dd (14.7, 5.8); 1.57, dd (14.6, 4.3)
9	32.1, CH	1.27, m	32.4, CH	1.13, m	72.9, C	-
10	29.6, CH_2_	1.27, m; 1.10, m	29.9, CH_2_	1.27, m; 1.09, m	35.3, CH	1.46, q (7.5)
11	11.2, CH_3_	0.82, t (7.3)	11.3, CH_3_	0.81, t (7.3)	8.2, CH_3_	0.88, t (7.5)
12	19.2, CH_3_	0.84, d (6.4)	19.2, CH_3_	0.83, d (6.2)	25.9, CH_3_	1.15, s
13	19.5, CH_3_	1.19, d (6.9)	19.3, CH_3_	1.16, d (7.0)	21.4, CH_3_	1.29, d (7.0)
14	8.3, CH_3_	1.97, s	9.8, CH_3_	2.00, s	56.1, CH_3_	3.87, s
15	-	-	8.8, CH_3_	2.02, s	8.5, CH_3_	1.90, s

**Table 2:** Antibacterial activities of compounds **1**–**4**.

Compounds	MIC (µg/mL)				
	*S. aureus*	*E. coli*	*B. subtilis*	MRSA	*M. tuberculosis*
1	64	32	64	128	128
2	64	64	64	128	128
3	8	64	16	128	64
4	64	64	32	128	128
Chloramphenicol	8	4	2	4	-
Ethambutol	-	-	-	-	8

## Supplementary Materials

The following are available online at https://www.mdpi.com/1660-3397/17/6/344/s1. HRESIMS, NMR, IR, UV of the new compounds **1**–**3**; ECD calculation data for **1**.
