# Supplementary material for: Production of New Antibacterial 4-Hydroxy-α-Pyrones by a Marine Fungus Aspergillus niger Cultivated in Solid Medium
Source: Mar Drugs. 2019 Jun 10;17(6):344. doi: 10.3390/md17060344 (PMC6627810; doi:10.3390/md17060344)
Supplement: Supplementary file 1 [file marinedrugs-17-00344-s001.pdf]

marine drugs

MDPI

Supplementary Material

Production of New Antibacterial 4-Hydroxy-*α*-Pyrones by a Marine Fungus *Aspergillus niger* Cultivated in Solid Medium

Lijian Ding [1], Lu Ren [1], Shuang Li [1], Jingjing Song [1], Zhiwen Han [1], Shan He [1,*] and Shihai Xu [2,*]

[1] Li Dak Sum Yip Yio Chin Kenneth Li Marine Biopharmaceutical Research Center, College of Food and Pharmaceutical Sciences, Ningbo University 315832, Ningbo, China; dinglijian@nbu.edu.cn (L.D.); renlurenlu@163.com (L.R.); lishuang9892@163.com (S.L.); 13123838771@163.com (J.S.); hanzhiwen1999@163.com (Z.H.)

[2] Department of Chemistry, College of Chemistry and Materials Science, Jinan University, Guangzhou 510632, China

List of Contents

No.	Content
1	Figure S1. IR spectrum of **1**
2	Figure S2. UV spectrum of compound **1**
3	Figure S3. ^{1}H NMR spectrum of compound **1** in $CDCl_3$
4	Figure S4. ^{13}C NMR spectrum of compound **1** in $CDCl_3$
5	Figure S5. DEPT 135 spectrum of compound **1** in $CDCl_3$
6	Figure S6. HSQC spectrum of compound **1** in $CDCl_3$
7	Figure S7. HMBC spectrum of compound **1** in $CDCl_3$
8	Figure S8. COSY spectrum of compound **1** in $CDCl_3$
9	Figure S9. NOESY spectrum of compound **1** in $CDCl_3$
10	Figure S10. HRESIMS of compound **2**
11	Figure S11. IR spectrum of compound **2**
12	Figure S12. UV spectrum of compound **2**
13	Figure S13. ^{1}H NMR spectrum of compound **2** in $CDCl_3$
14	Figure S14. ^{13}C NMR spectrum of compound **2** in $CDCl_3$
15	Figure S15. DEPT 135 spectrum of compound **2** in $CDCl_3$
16	Figure S16. HSQC spectrum of compound **2** in $CDCl_3$
17	Figure S17. HMBC spectrum of compound **2** in $CDCl_3$
18	Figure S18. COSY spectrum of compound **2** in $CDCl_3$
19	Figure S19. NOESY spectrum of compound **2** in $CDCl_3$
20	Figure S20. HRESIMS of compound **2**
21	Figure S21. IR spectrum of compound **3**
22	Figure S22. UV spectrum of compound **3**
23	Figure S23. ^{1}H NMR spectrum of compound **3** in $CDCl_3$
24	Figure S24. ^{13}C NMR spectrum of compound **3** in $CDCl_3$
25	Figure S25. DEPT 135 spectrum of compound **3**in $CDCl_3$
26	Figure S26. HSQC spectrum of compound **3** in $CDCl_3$
27	Figure S27. HMBC spectrum of compound **3** in $CDCl_3$
28	Figure S28. COSY spectrum of compound **3** in $CDCl_3$
29	Figure S29. NOESY spectrum of compound **3** in $CDCl_3$
30	Figure S30. HRESIMS of compound **3**
31	S1. ECD calculation details

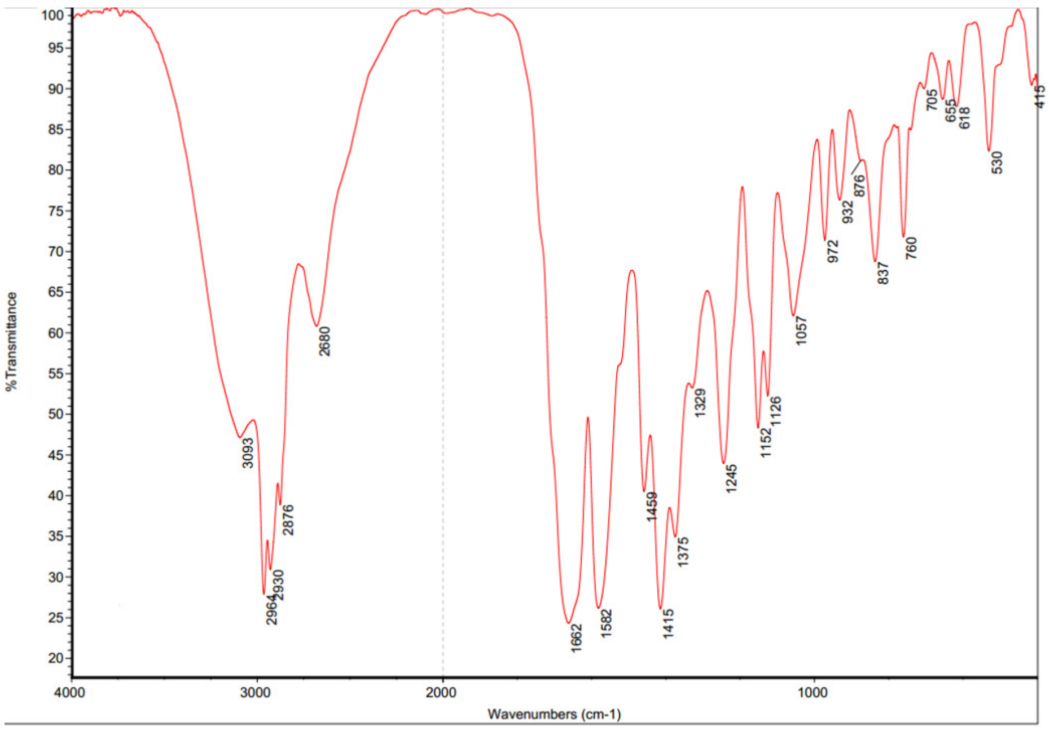

Figure S1. IR spectrum of compound **1**

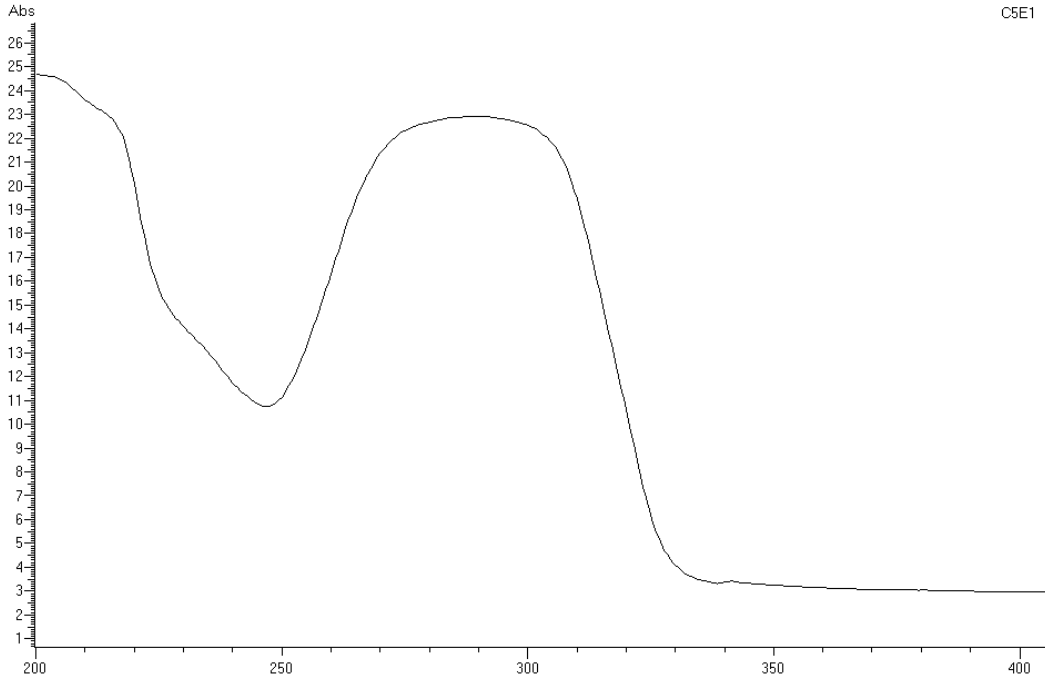

Figure S2. UV spectrum of compound **1**

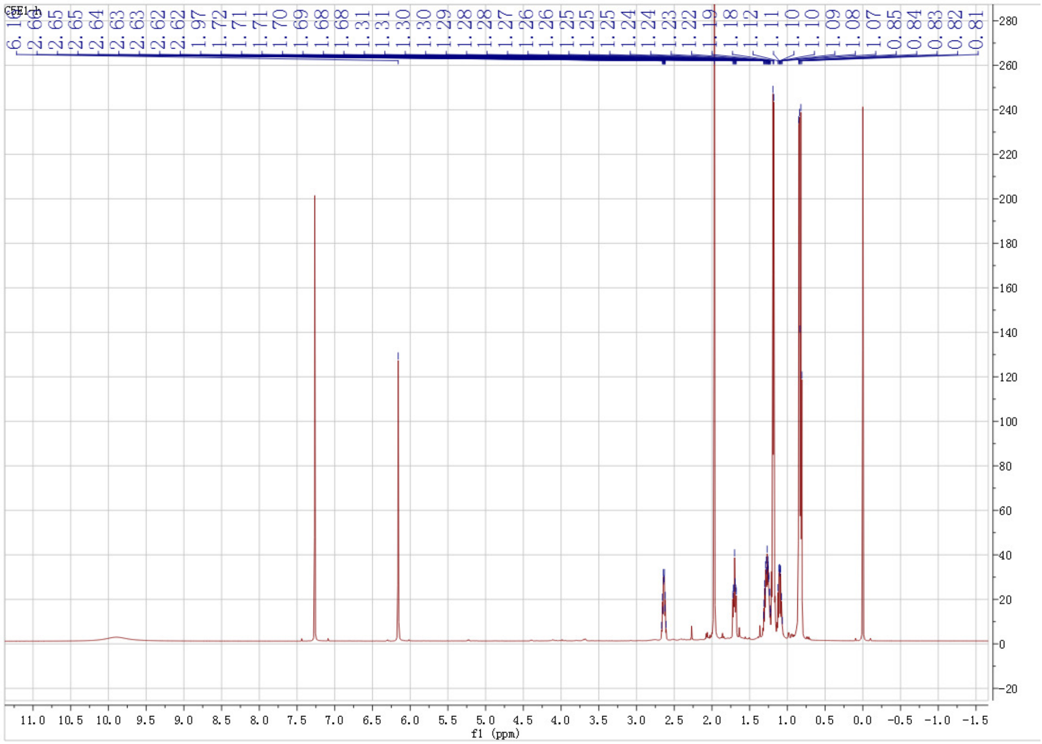

Figure S3. ^{1}H NMR spectrum of compound **1** in $CDCl_3$

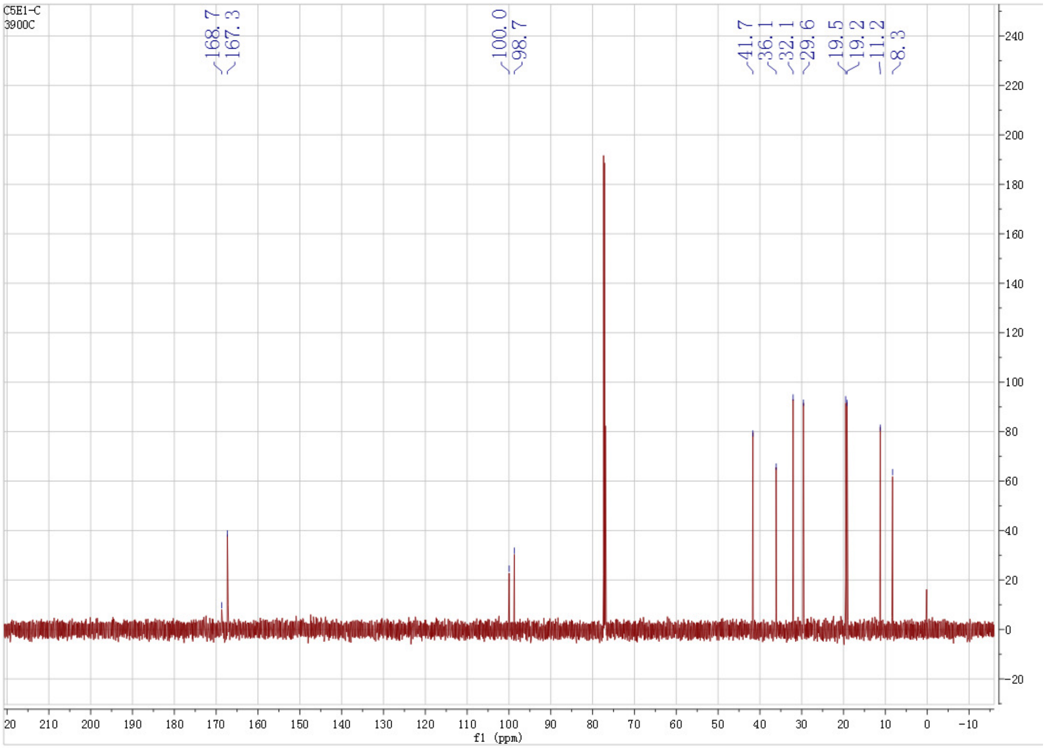

Figure S4. ^{13}C NMR spectrum of compound **1** in $CDCl_3$

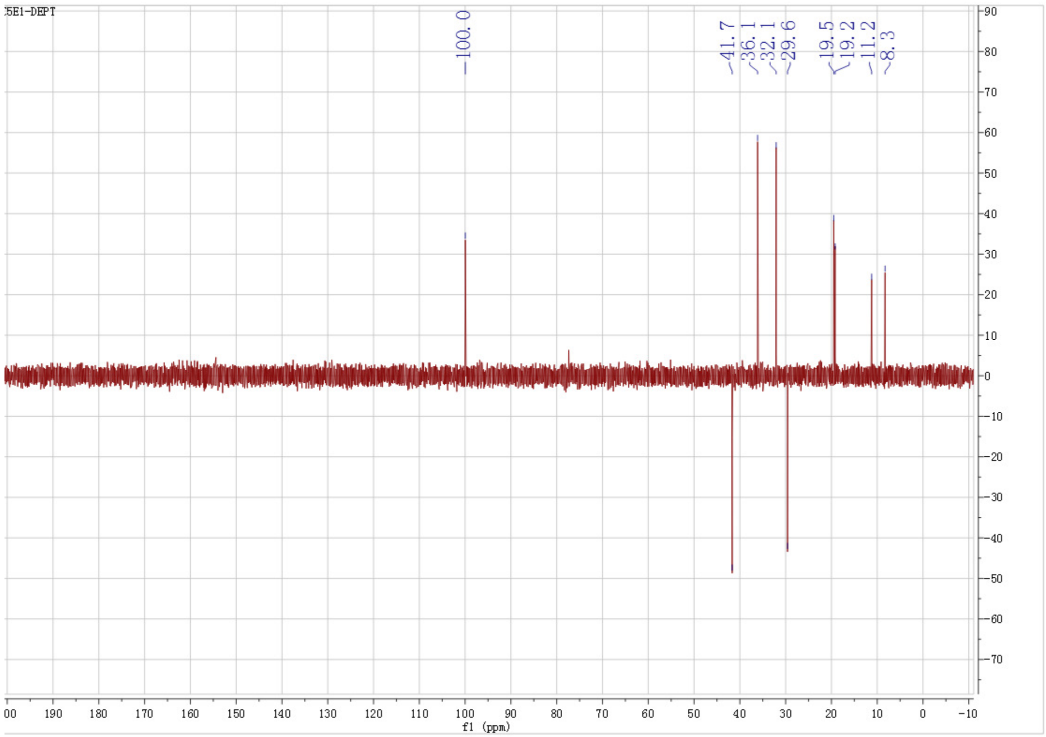

Figure S5. DEPT spectrum of compound 1 in $CDCl_3$

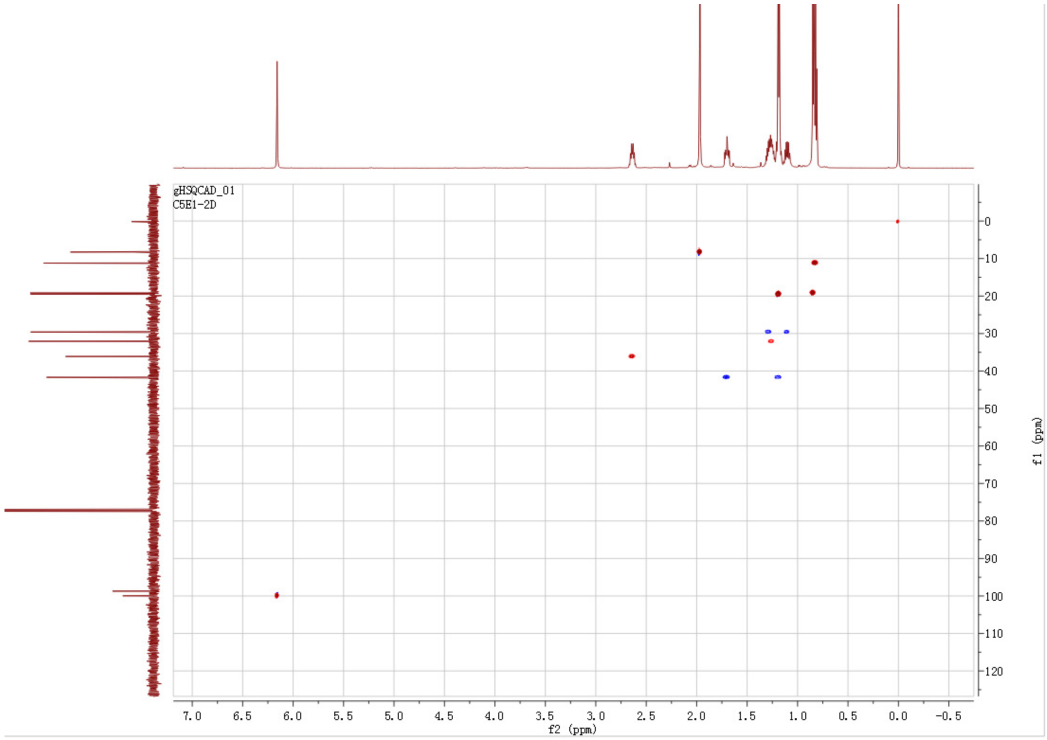

Figure S6. HSQC spectrum of compound **1** in $CDCl_3$

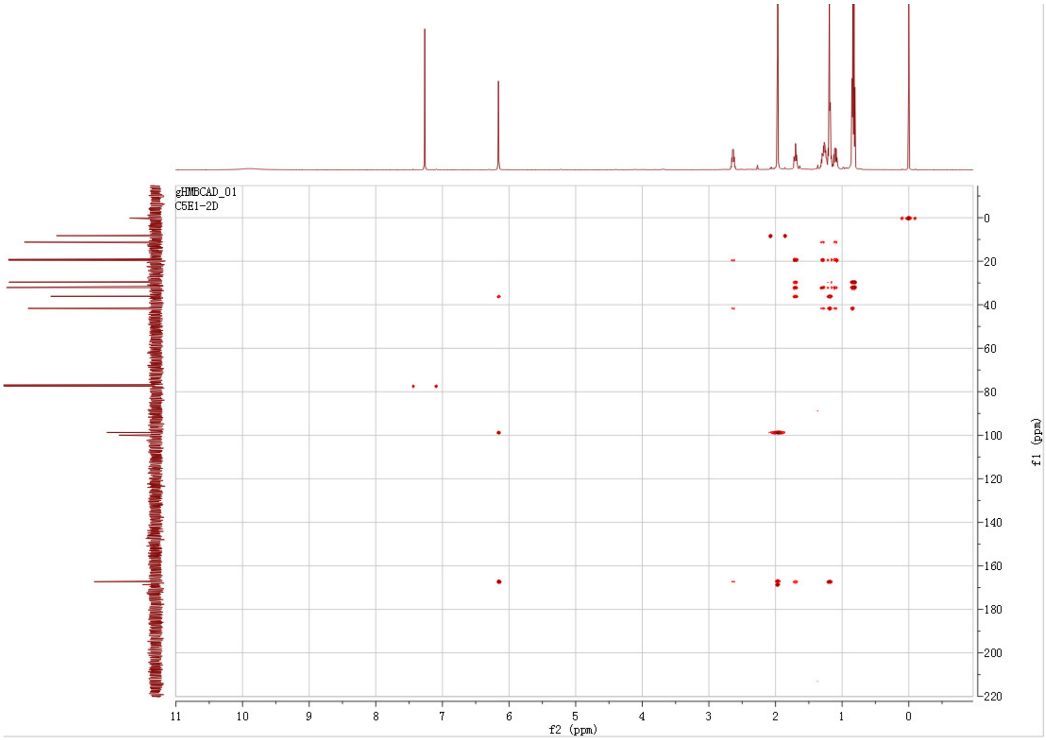

Figure S7. HMBC spectrum of compound **1** in $CDCl_3$

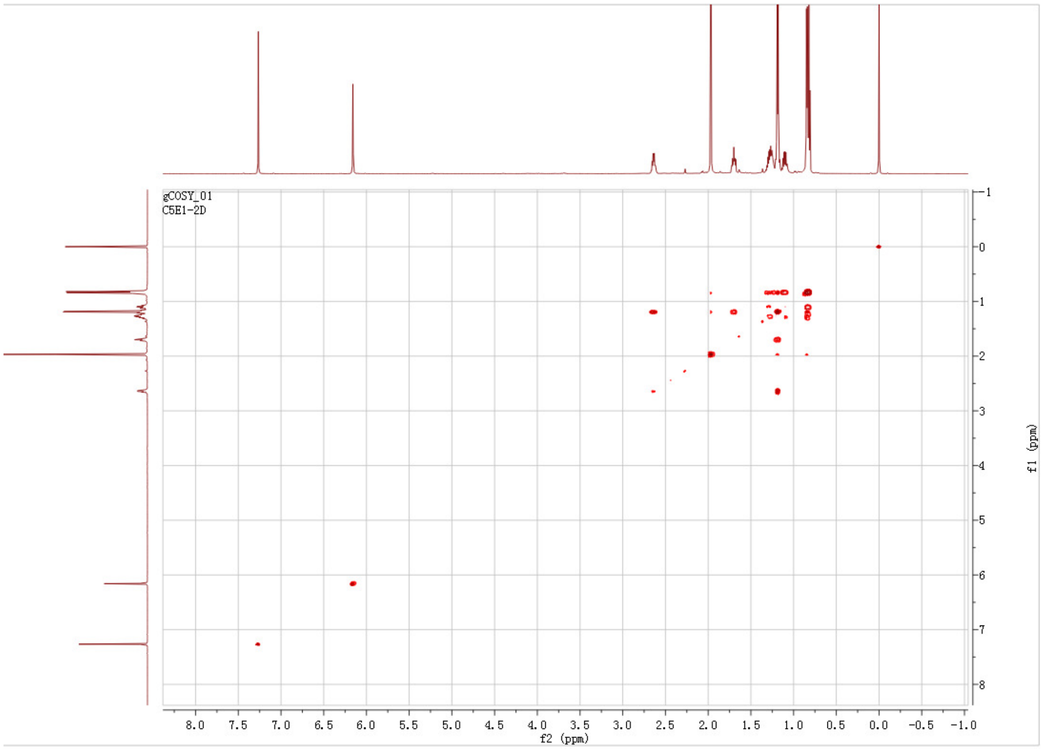

Figure S8. COSY spectrum of compound **1** in $CDCl_3$

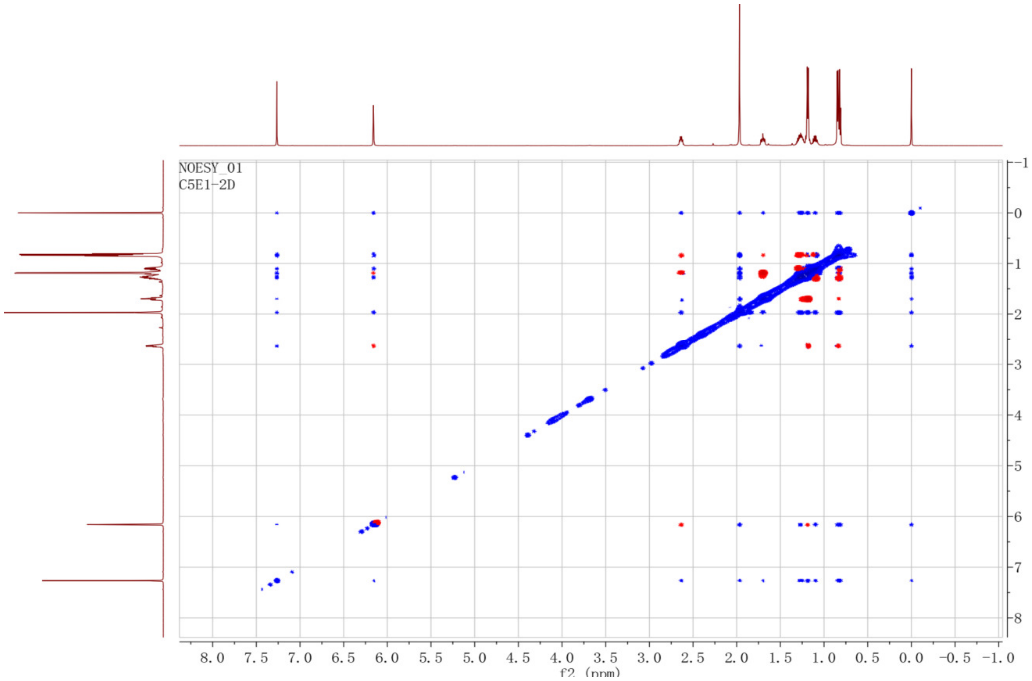

Figure S9. NOESY spectrum of compound **1** in $CDCl_3$

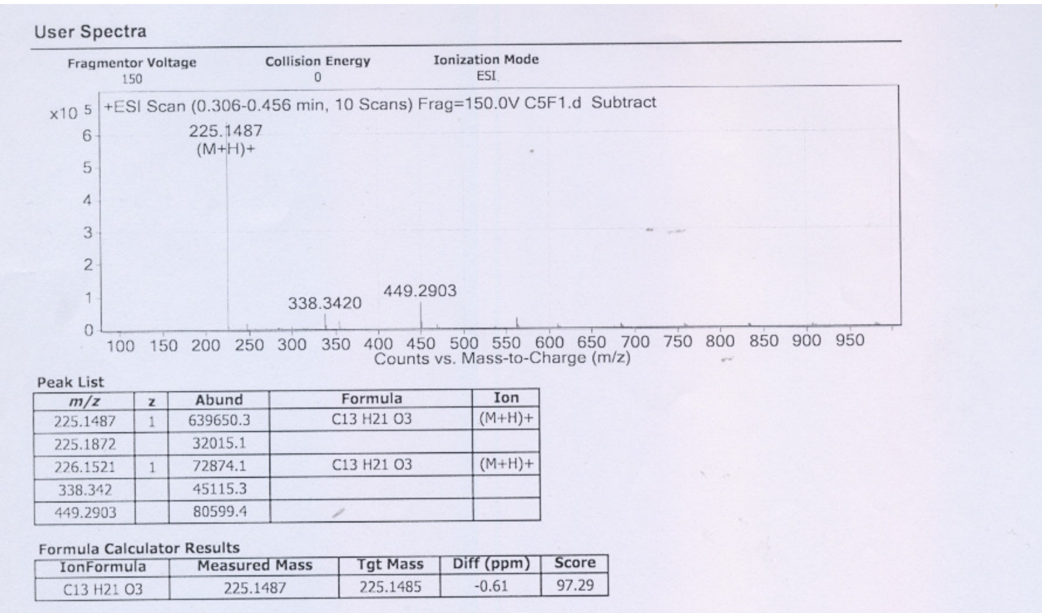

Peak List

m/z	z	Abund	Formula	Ion
225.1487	1	639650.3	C13 H21 O3	(M+H)+
225.1872		32015.1		
226.1521	1	72874.1	C13 H21 O3	(M+H)+
338.342		45115.3		
449.2903		80599.4		

Formula Calculator Results

IonFormula	Measured Mass	Tgt Mass	Diff (ppm)	Score
C13 H21 O3	225.1487	225.1485	-0.61	97.29

Figure S10. HRESIMS of compound **1**

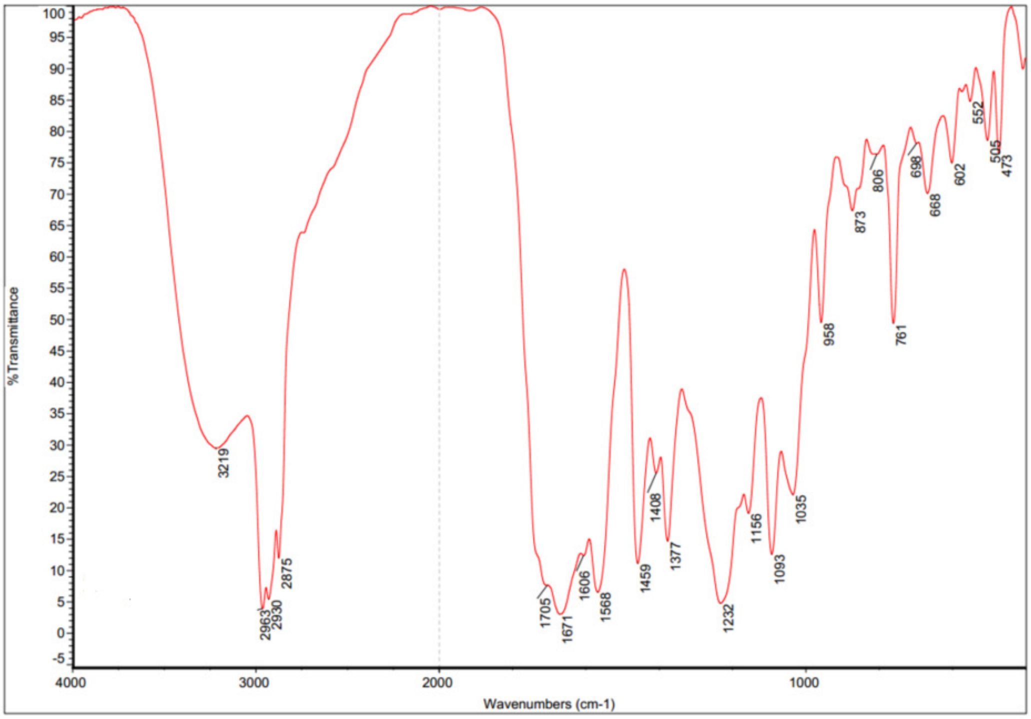

Figure S11. IR spectrum of compound **2**

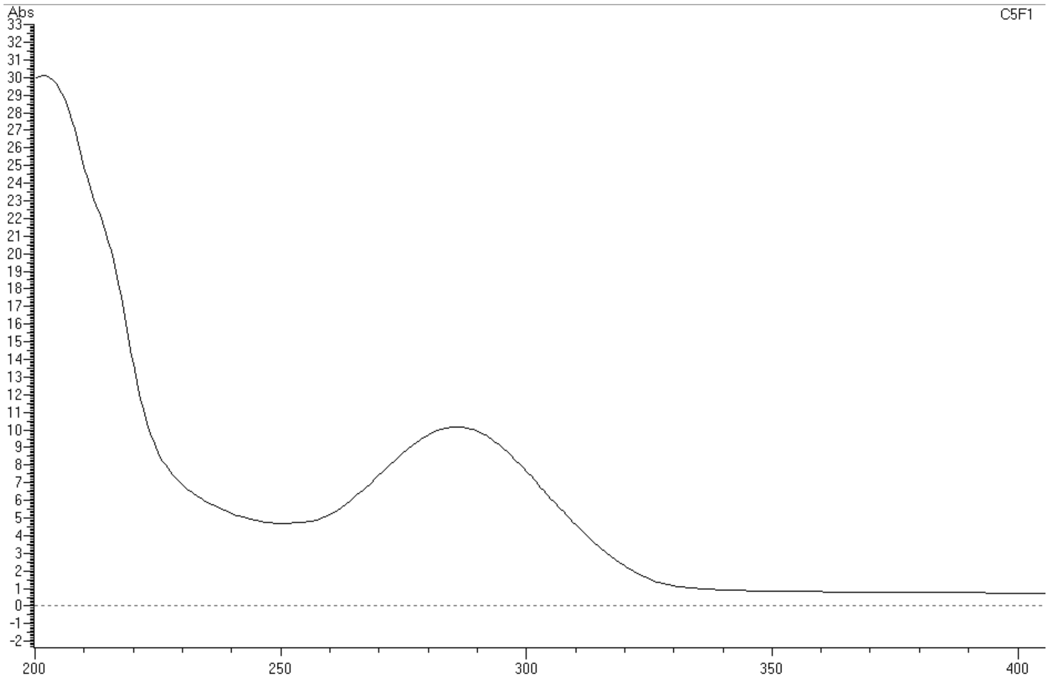

Figure S12. UV spectrum of compound **2**

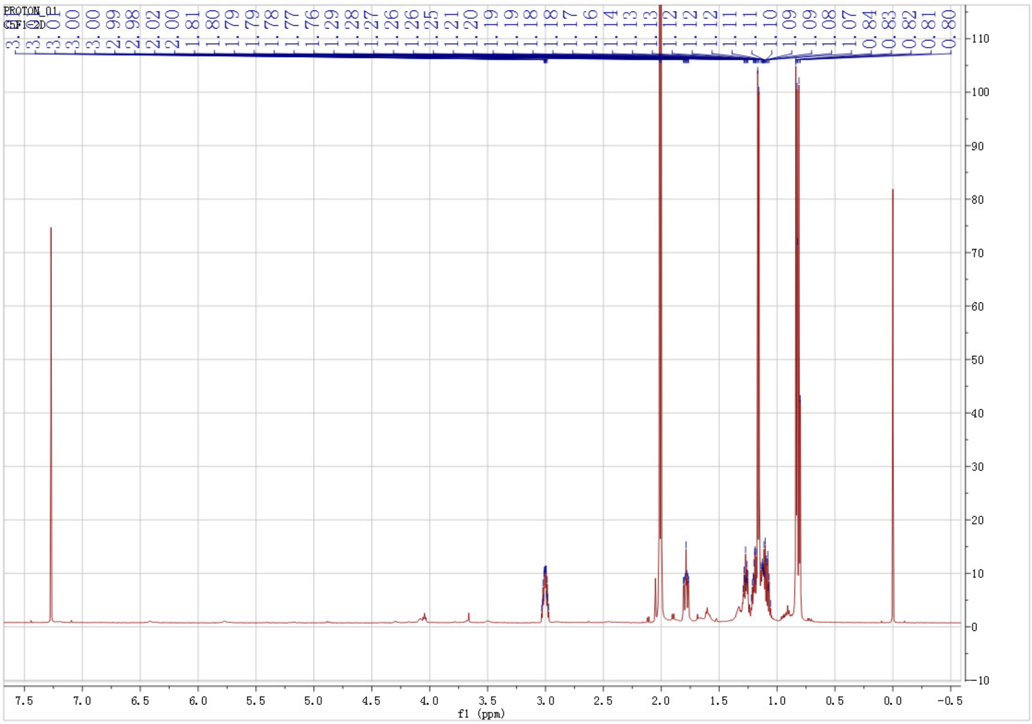

Figure S13. ^{1}H NMR spectrum of compound **2** in $CDCl_3$

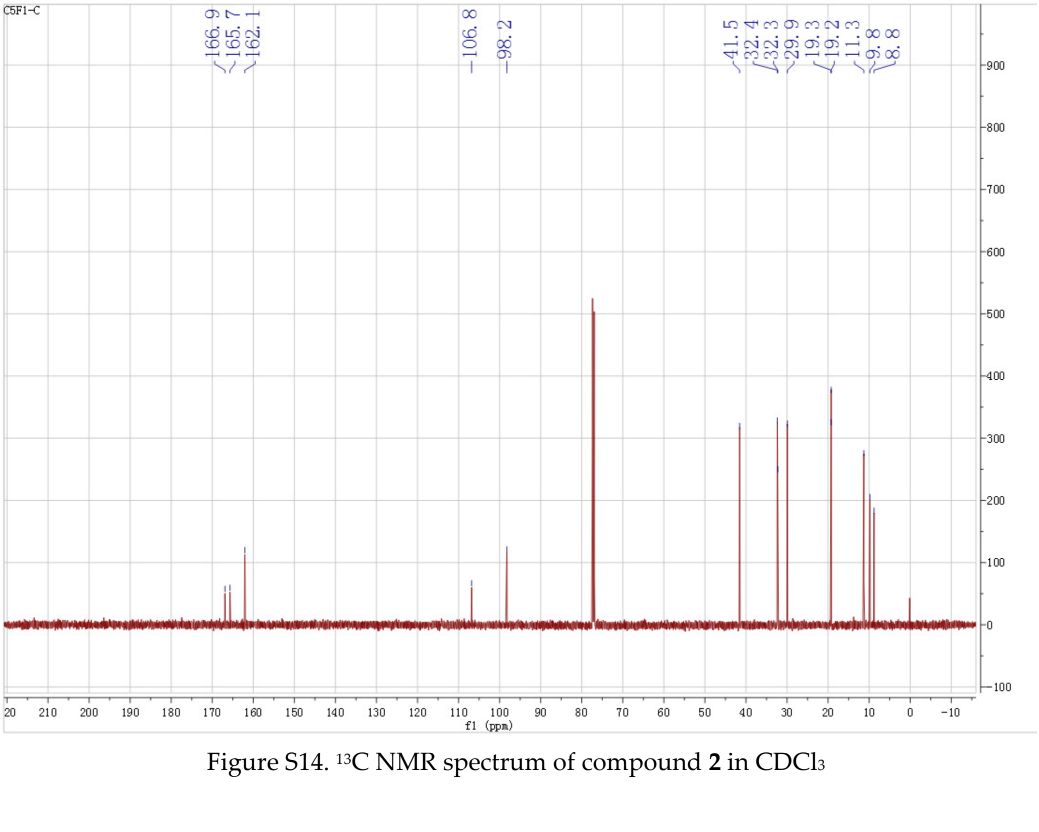

Figure S14. ^{13}C NMR spectrum of compound **2** in $CDCl_3$

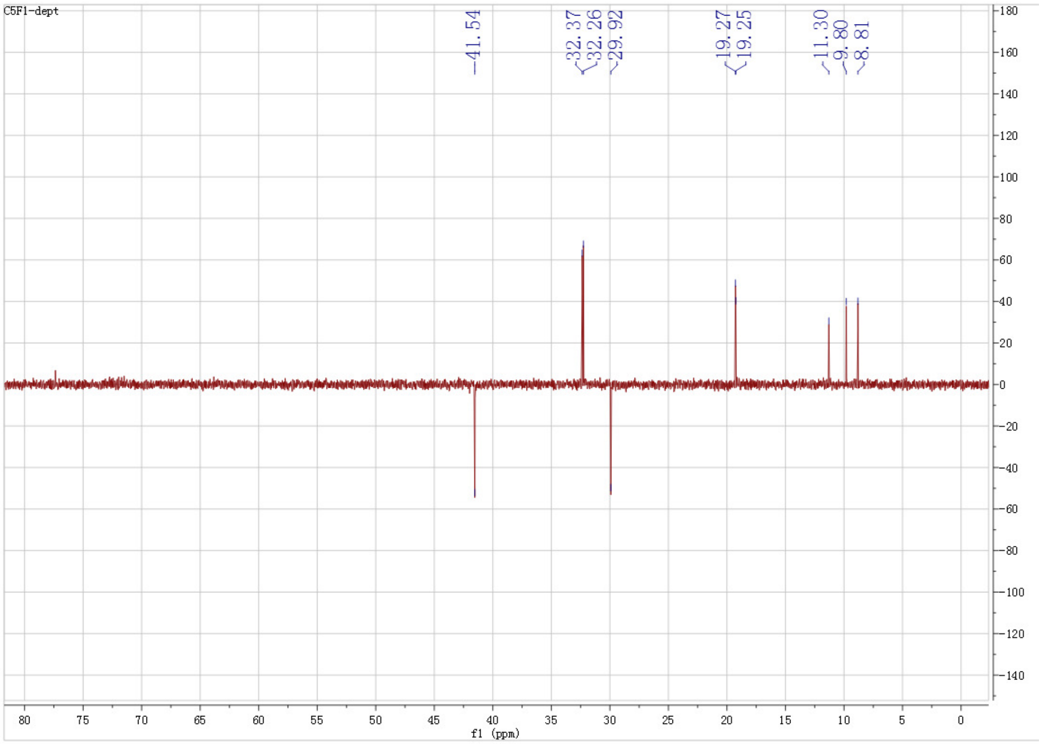

Figure S15. DEPT 135 spectrum of compound **2** in $CDCl_3$

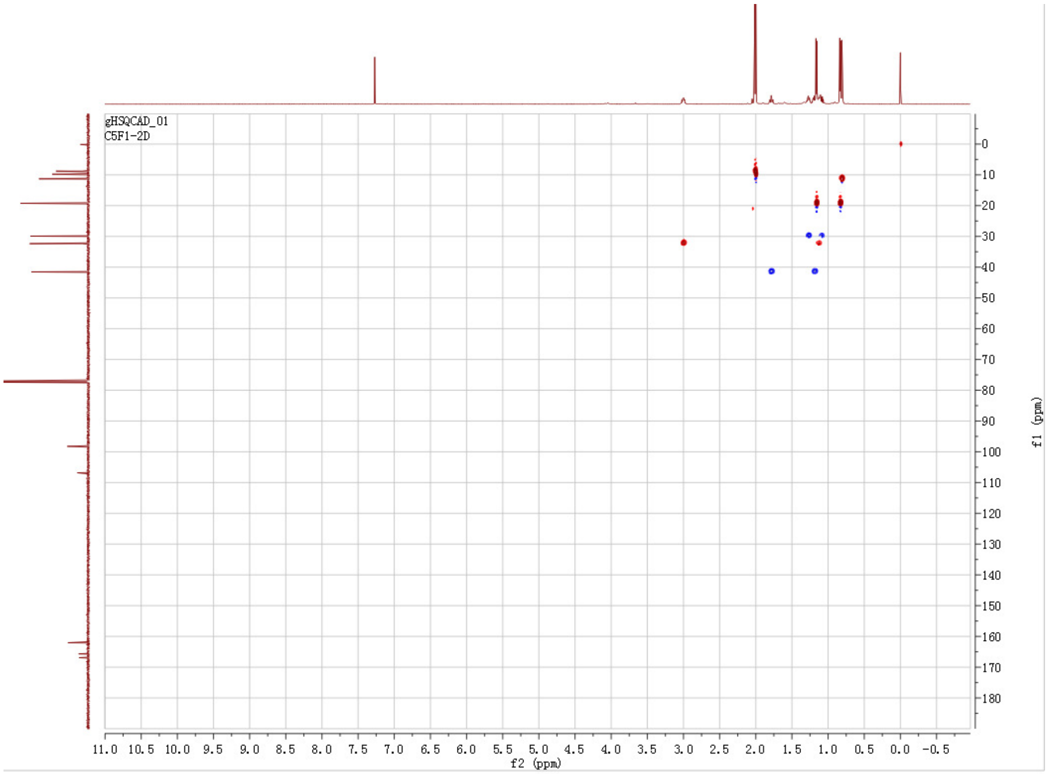

Figure S16. HSQC spectrum of compound **2** in $CDCl_3$

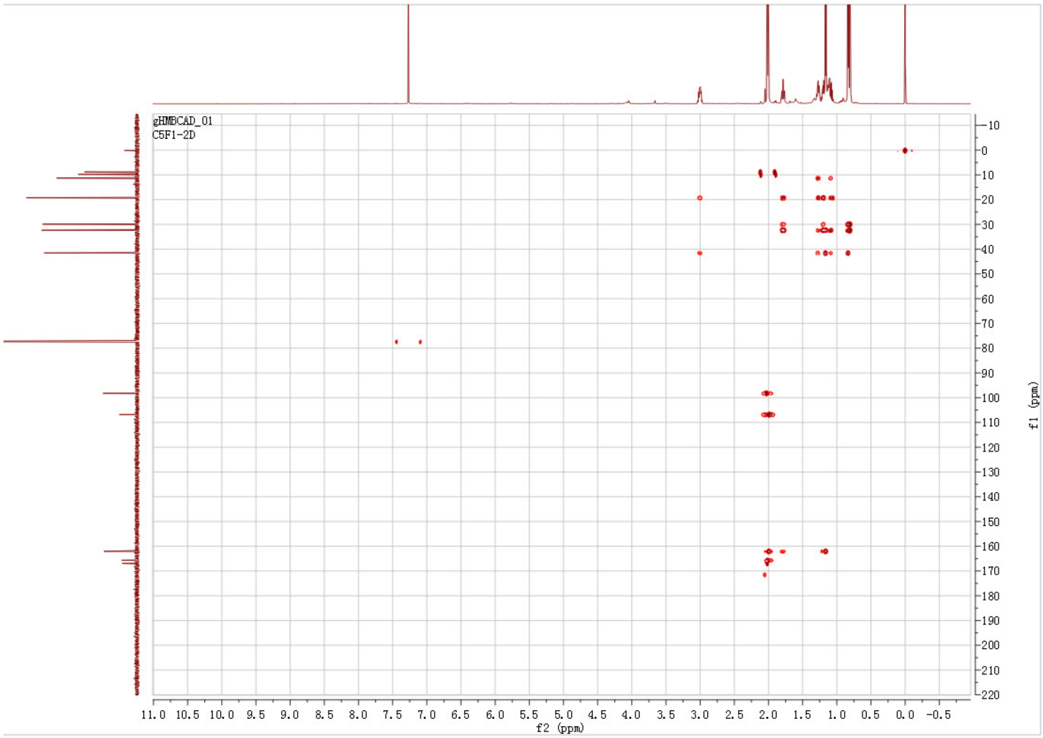

Figure S17. HMBC spectrum of compound **2** in $CDCl_3$

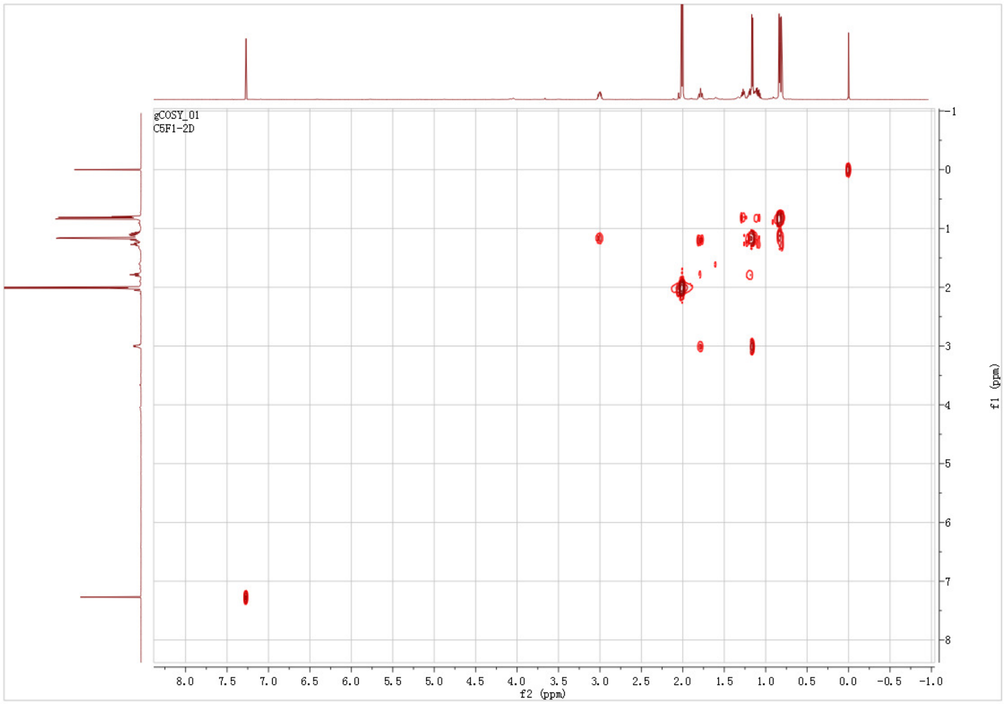

Figure S18. COSY spectrum of compound **2** in $CDCl_3$

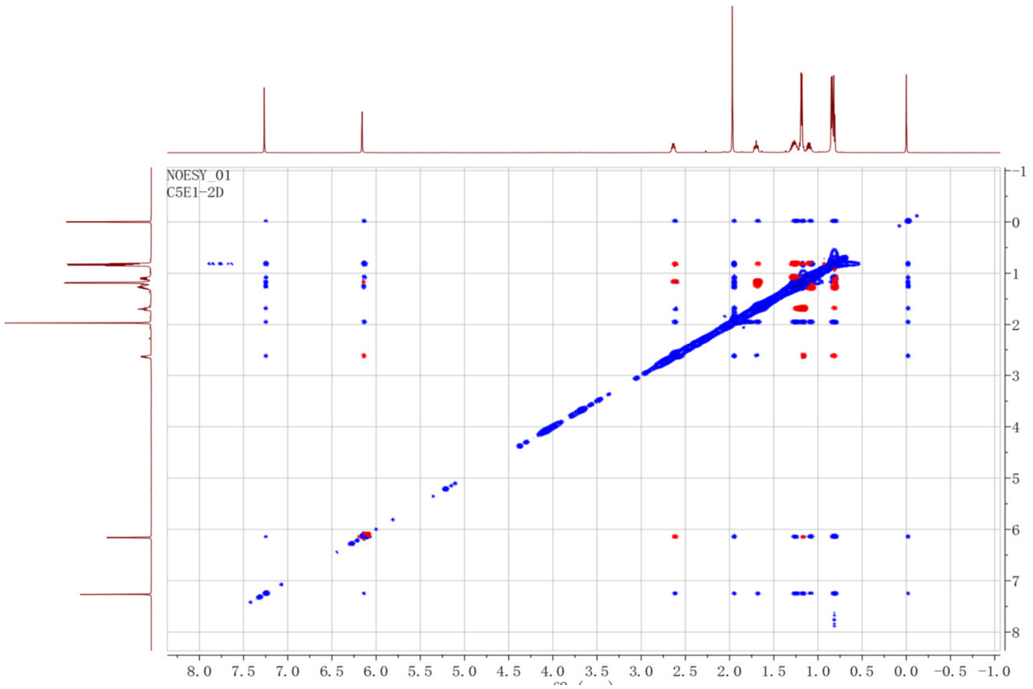

Figure S19. NOESY spectrum of compound **2** in $CDCl_3$

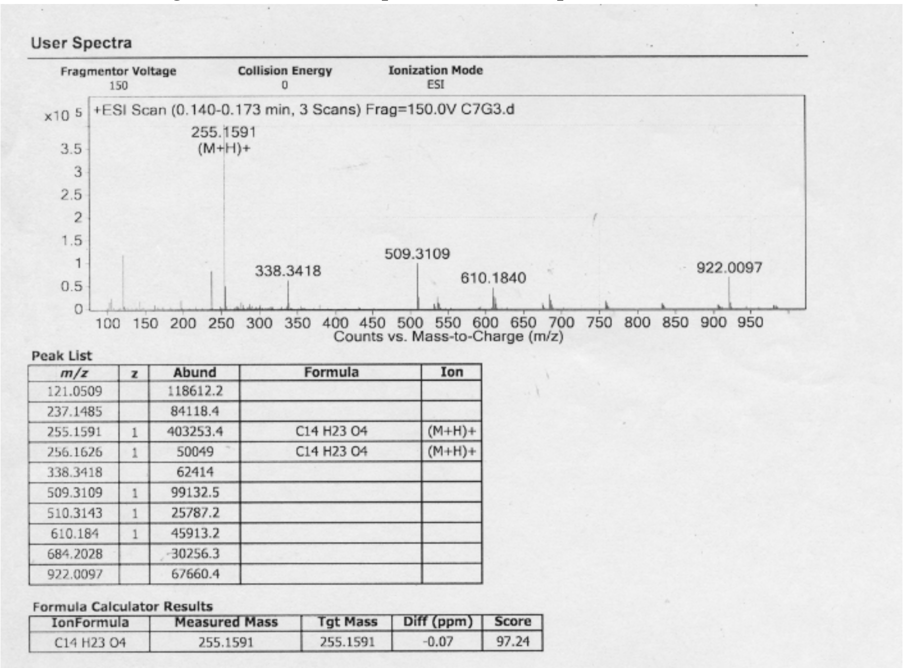

User Spectra

Fragmentor Voltage	Collision Energy	Ionization Mode
150	0	ESI

Peak List

m/z	z	Abund	Formula	Ion
121.0509		118612.2		
237.1485		84118.4		
255.1591	1	403253.4	C14 H23 O4	(M+H)+
256.1626	1	50049	C14 H23 O4	(M+H)+
338.3418		62414		
509.3109	1	99132.5		
510.3143	1	25787.2		
610.184	1	45913.2		
684.2028		30256.3		
922.0097		67660.4		

Formula Calculator Results

IonFormula	Measured Mass	Tgt Mass	Diff (ppm)	Score
C14 H23 O4	255.1591	255.1591	-0.07	97.24

Figure S20. HRESIMS of compound **2**

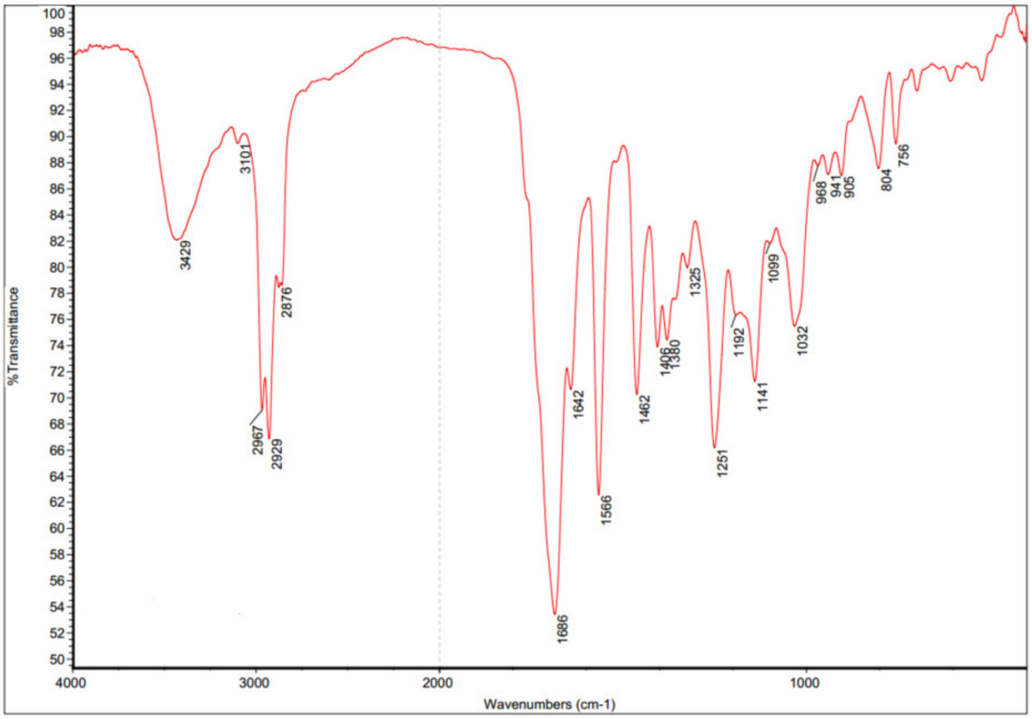

Figure S21. IR spectrum of compound **3**

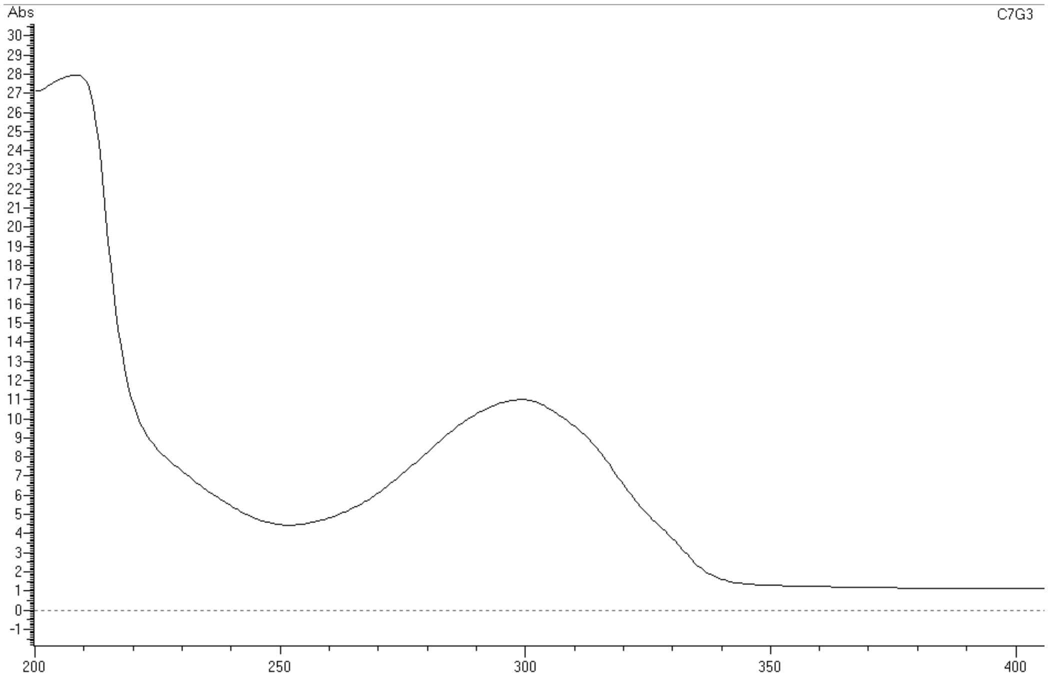

Figure S22. UV spectrum of compound **3**

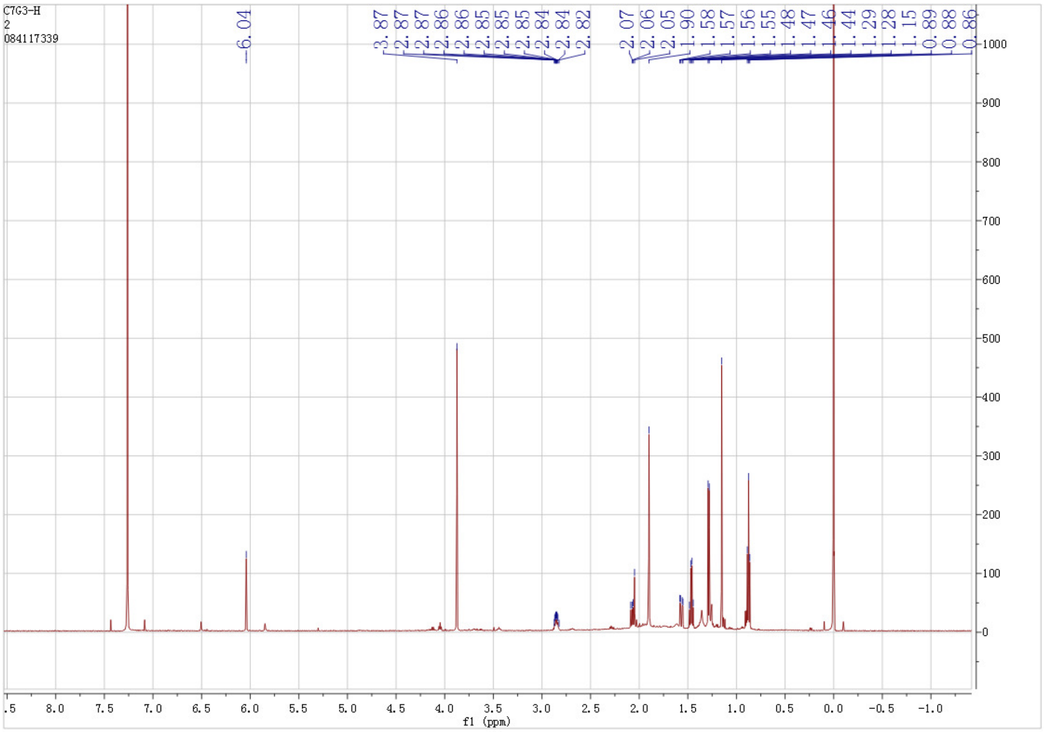

Figure S23. ¹H NMR spectrum of compound **3** in $CDCl_3$

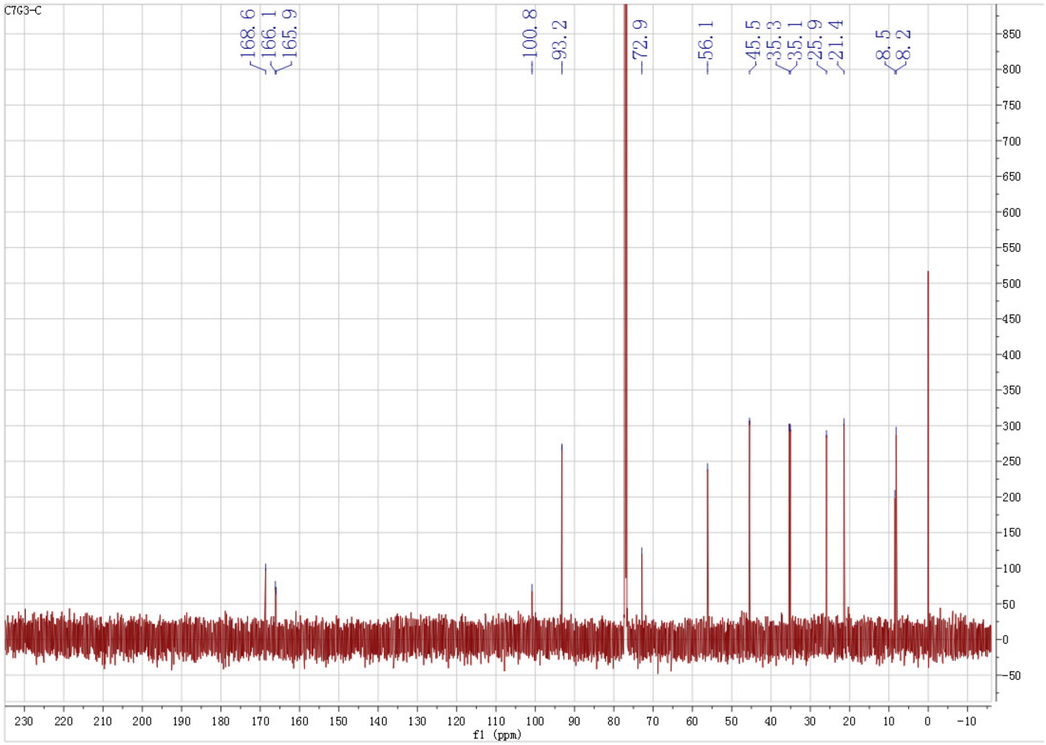

Figure S24. ¹³C NMR spectrum of compound **3** in $CDCl_3$

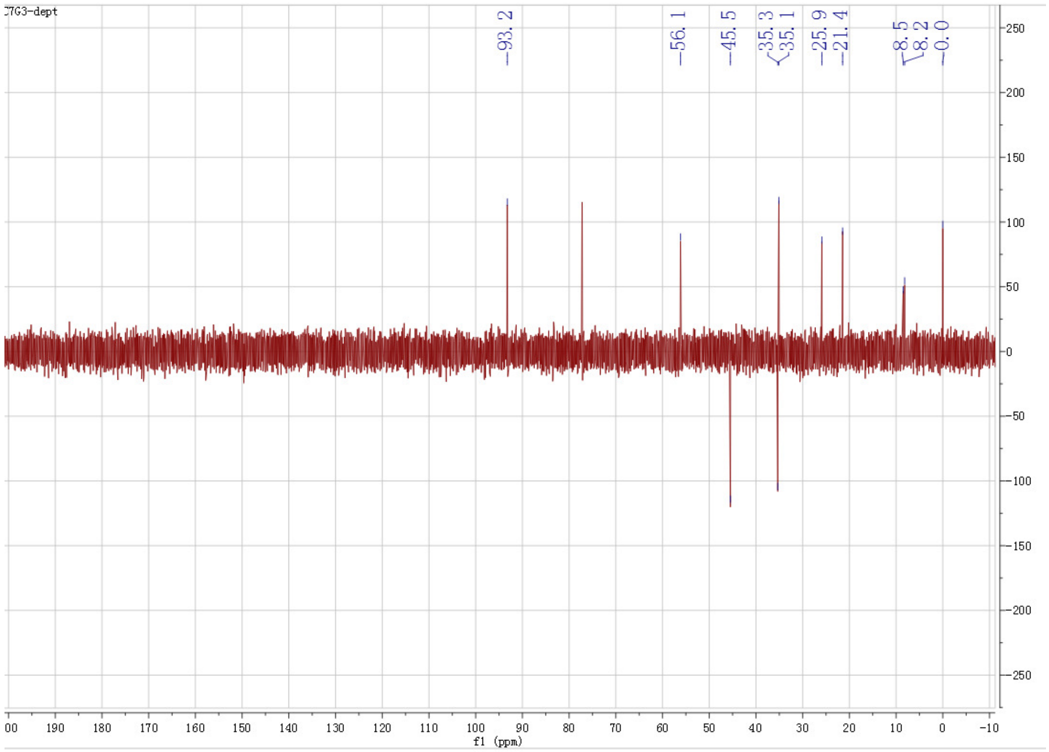

Figure S25. DEPT 135 spectrum of compound **3** in $CDCl_3$

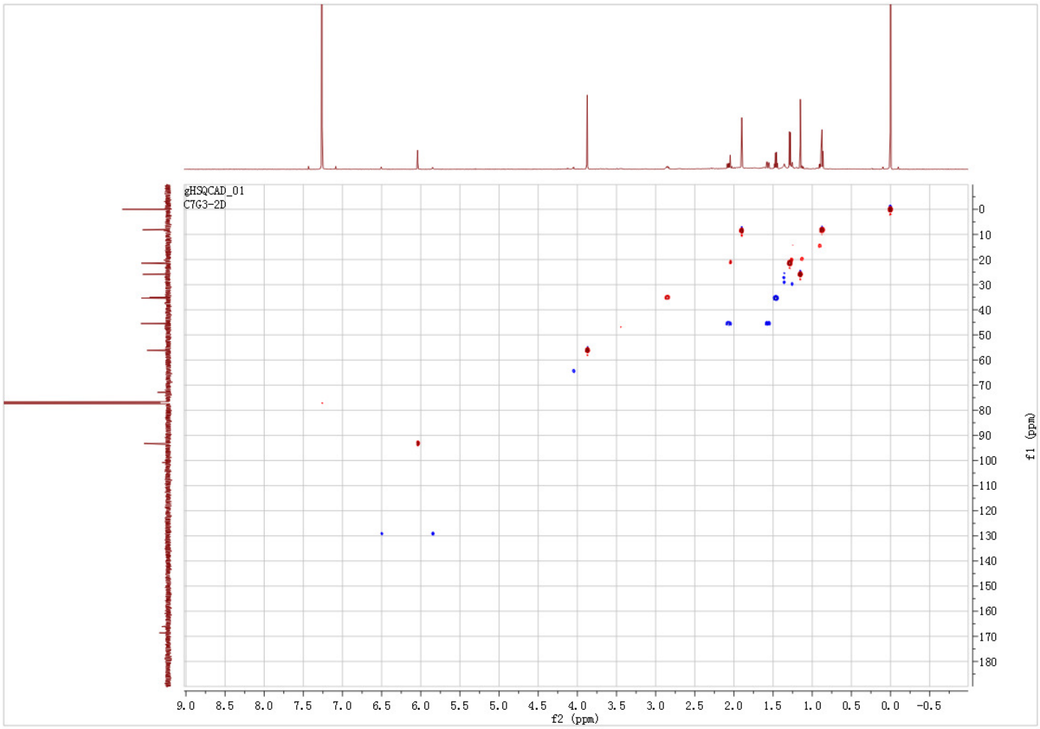

Figure S26. HSQC spectrum of compound **3** in $CDCl_3$

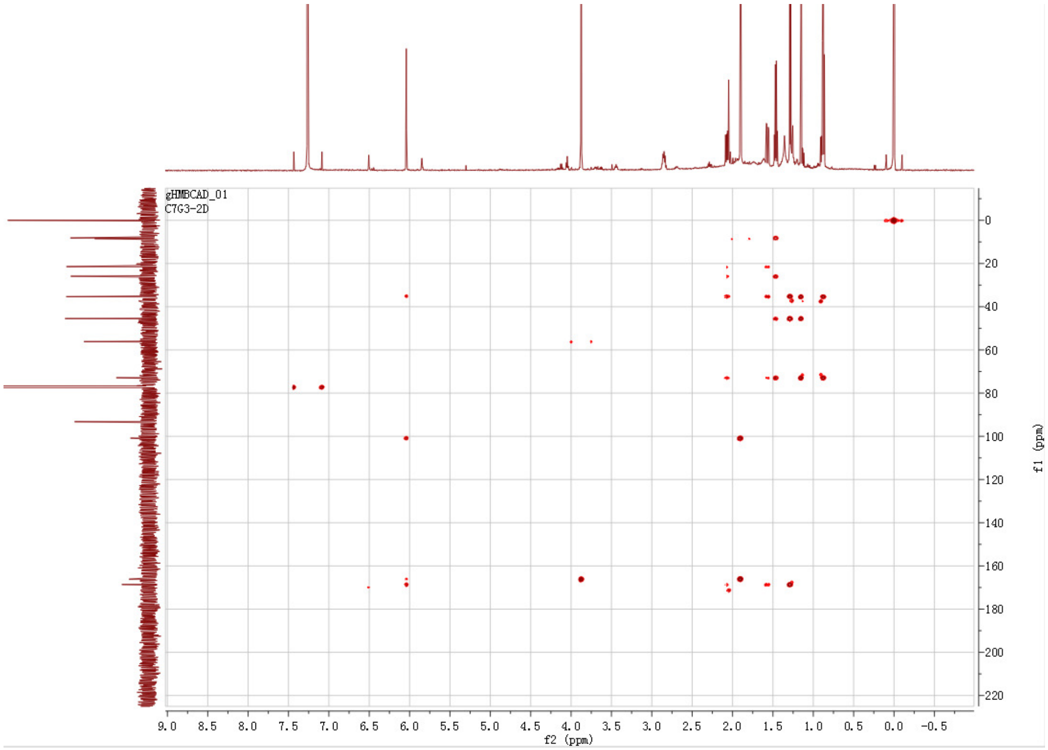

Figure S27 HMBC spectrum of compound **3** in $CDCl_3$

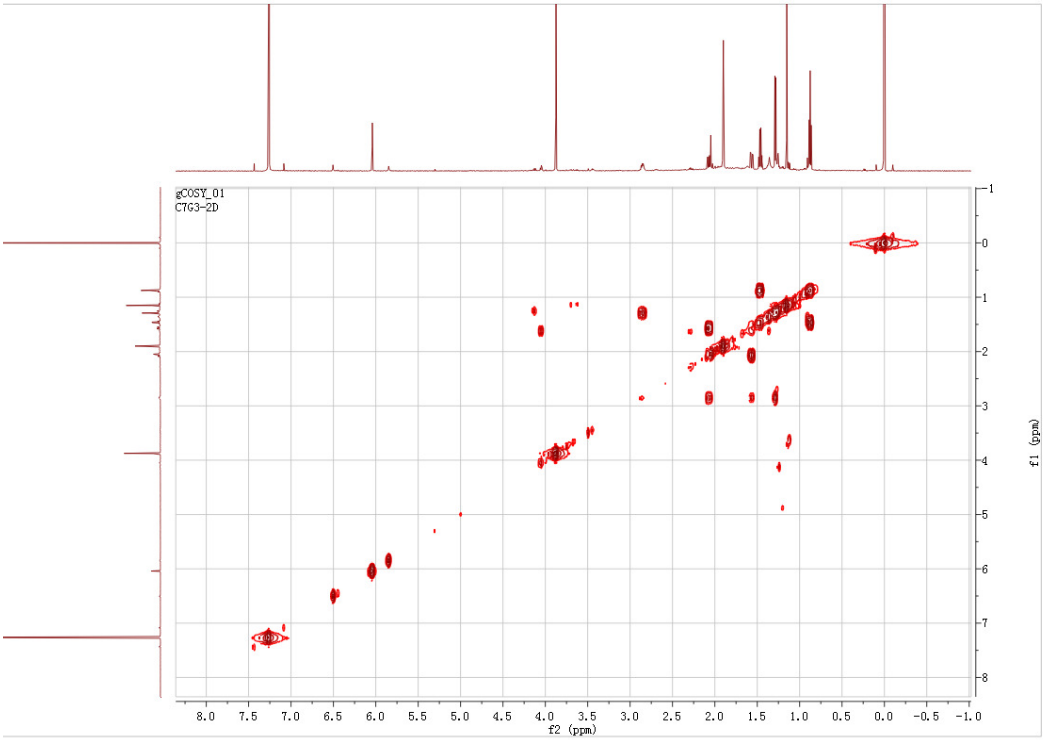

Figure S28. COSY spectrum of compound **3** in $CDCl_3$

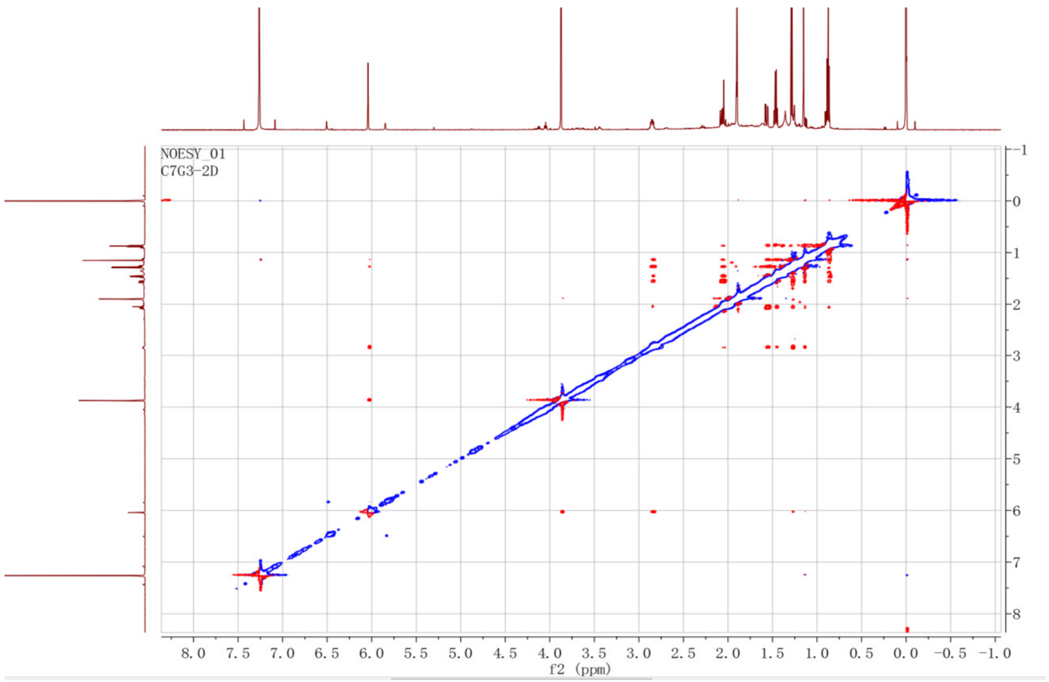

Figure S20. NOESY spectrum of compound **3** in $CDCl_3$

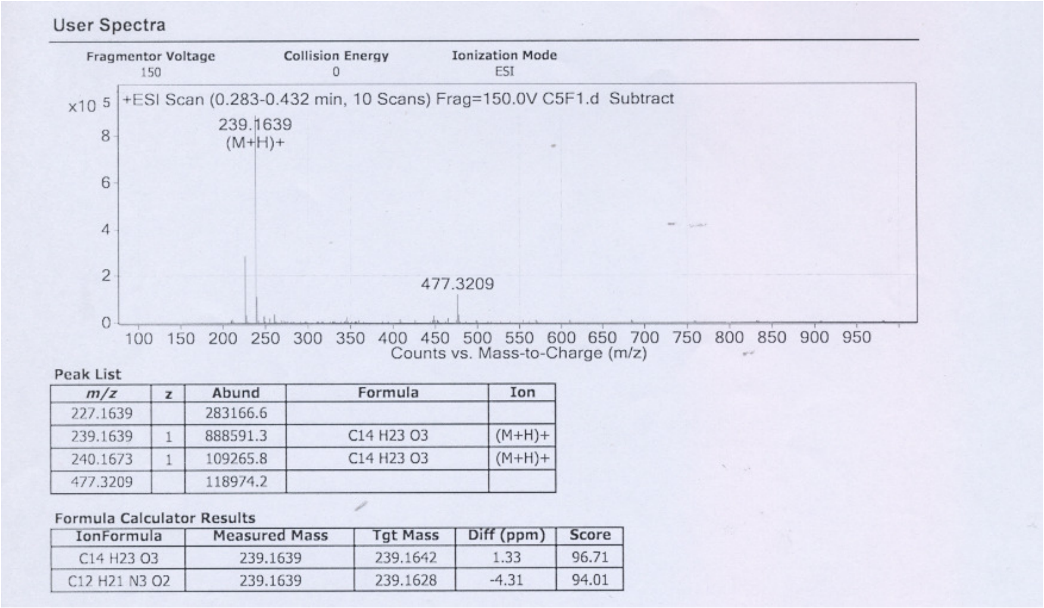

User Spectra

Fragmentor Voltage	Collision Energy	Ionization Mode
150	0	ESI

Peak List

m/z	z	Abund	Formula	Ion
227.1639		283166.6		
239.1639	1	888591.3	C14 H23 O3	(M+H)+
240.1673	1	109265.8	C14 H23 O3	(M+H)+
477.3209		118974.2		

Formula Calculator Results

IonFormula	Measured Mass	Tgt Mass	Diff (ppm)	Score
C14 H23 O3	239.1639	239.1642	1.33	96.71
C12 H21 N3 O2	239.1639	239.1628	-4.31	94.01

Figure S30. HRESIMS of compound **3**

S1. ECD calculation details

1. Methods

In general, conformational analyses were carried out via random searching in the Sybyl-X 2.0 using the MMFF94S force field with an energy cutoff of 2.5 kcal/mol [1]. The results showed eight lowest energy conformer for both compounds. Subsequently, the conformers were re-optimized using DFT at the b3lyp/6-311+g(d, p) level in MeOH using the polarizable conductor calculation model (CPCM) by the GAUSSIAN 09 program [2]. The energies, oscillator strengths, and rotational strengths (velocity) of the first 50 electronic excitations were calculated using the TDDFT methodology at the b3lyp/6-311+g(d,p) level in MeOH. The ECD spectra were simulated by the overlapping Gaussian function (half the bandwidth at 1/e peak height, sigma = 0.30 for all) [3]. To get the final spectra, the simulated spectra of the conformers were averaged according to the Boltzmann distribution theory and their relative Gibbs free energy (ΔG). The Chem3D 8.0.3 software package was used for visualization of the results.

2. Results

Table S1. Gibbs free energies[a] and equilibrium populations[b] of low-energy conformers of **1**.

**Conformers**	**ΔG**	**P(%)/100**
1a	0.00073	31.58
1b	0.0	68.42

[a]B3LYP/6-311+G(d,p), in kcal/mol.

[b]From ΔG values at 298.15K.

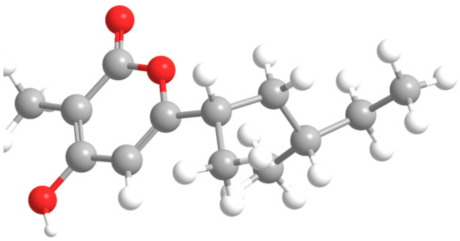

1a

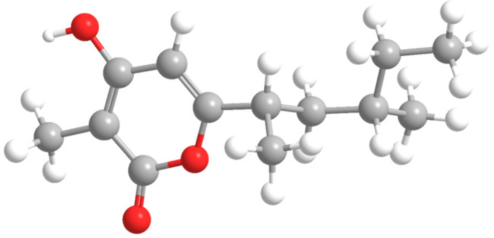

1b

Figure S31 low-energy conformers of **1**.

Table S2. Cartesian coordinates for the low-energy reoptimized random reseach conformers of **1** at B3LYP/6-311+G(d,p) level of theory in CH_3OH.

**1a**		**Standard Orientation (Ångstroms)**			
**Center number**	**Atomic number**	**Atomic Type**	**X**	**Y**	**Z**
1	6	0	-1.271164	1.386835	-0.01648
2	6	0	-0.365513	0.621908	-0.648268
3	8	0	-0.644614	-0.663807	-0.938605
4	6	0	-1.830949	-1.273866	-0.594239
5	6	0	-2.809139	-0.499182	0.093017
6	6	0	-2.519169	0.808598	0.365606
7	6	0	-4.093322	-1.173319	0.467358
8	8	0	-1.935059	-2.445541	-0.915723
9	6	0	0.993054	1.02876	-1.142865
10	6	0	2.105955	0.029198	-0.760309
11	6	0	1.345983	2.467037	-0.783747
12	6	0	2.609311	0.038248	0.688733
13	6	0	3.698561	-1.025964	0.884585
14	6	0	4.934158	-0.869871	0.004798
15	6	0	1.496042	-0.180564	1.712867
16	8	0	-3.427227	1.551412	1.010376
17	1	0	-1.056309	2.418218	0.225122
18	1	0	-3.907787	-2.027395	1.124648
19	1	0	-4.764041	-0.486363	0.981265
20	1	0	-4.605731	-1.555312	-0.419901
21	1	0	0.910562	0.968161	-2.235612
22	1	0	2.94638	0.252366	-1.422249
23	1	0	1.77656	-0.983412	-1.013777
24	1	0	1.377478	2.622398	0.297253
25	1	0	2.331108	2.709948	-1.187217
26	1	0	0.629281	3.17356	-1.208177

27	1	0	3.067565	1.015002	0.888597
28	1	0	4.008799	-1.000733	1.934661
29	1	0	3.254466	-2.015893	0.721082
30	1	0	4.71593	-1.042698	-1.051498
31	1	0	5.361151	0.133779	0.099189
32	1	0	5.706659	-1.585742	0.297486
33	1	0	0.932365	-1.093633	1.492671
34	1	0	1.9167	-0.283426	2.717306
35	1	0	0.789612	0.650575	1.737723
36	1	0	-3.098394	2.447173	1.146718

1b		Standard Orientation (Ångstroms)			
Center number	Atomic number	Atomic Type	X	Y	Z
1	6	0	-1.270774	1.351917	0.134723
2	6	0	-0.540938	0.254406	0.38331
3	8	0	-1.104994	-0.970208	0.348251
4	6	0	-2.439475	-1.172019	0.07004
5	6	0	-3.243656	-0.030072	-0.228683
6	6	0	-2.65496	1.203335	-0.174036
7	6	0	-4.691784	-0.25393	-0.548253
8	8	0	-2.814429	-2.331093	0.104198
9	6	0	0.920462	0.223858	0.705044
10	6	0	1.690136	-0.390684	-0.47777
11	6	0	1.174824	-0.508254	2.025048
12	6	0	3.21312	-0.469498	-0.319755
13	6	0	3.829415	0.900409	-0.016784
14	6	0	5.347998	0.891859	0.121887
15	6	0	3.810453	-1.100942	-1.576768
16	8	0	-3.303983	2.351141	-0.396458
17	1	0	-0.822867	2.334678	0.169903
18	1	0	-4.82528	-1.227927	-1.01969
19	1	0	-5.314819	-0.232288	0.351002
20	1	0	-5.077497	0.497494	-1.242464
21	1	0	1.215916	1.268821	0.819791
22	1	0	1.461356	0.192479	-1.378127
23	1	0	1.304065	-1.40054	-0.65245
24	1	0	0.537065	-0.113941	2.819879
25	1	0	0.978783	-1.578714	1.926376
26	1	0	2.213083	-0.380724	2.336126
27	1	0	3.439969	-1.132407	0.52409
28	1	0	3.536666	1.603742	-0.806648
29	1	0	3.401661	1.2882	0.912866
30	1	0	5.711442	1.862295	0.47035
31	1	0	5.672488	0.138044	0.846247
32	1	0	5.845629	0.679331	-0.827159
33	1	0	4.877632	-1.302189	-1.462637
34	1	0	3.319487	-2.050901	-1.805836
35	1	0	3.682627	-0.441382	-2.44205
36	1	0	-4.239083	2.194885	-0.575525

Reference

[1] Sybyl Software, version X 2.0; Tripos Associates Inc.: St. Louis, MO, **2013**.

[2] Frisch, M.J.; Trucks, G.W.; Schlegel, H.B.; Scuseria, G.E.; Robb, M.A.; Cheeseman, J.R.;

Scalmani, G.; Barone, V.; Mennucci, B.; Petersson, G.A.; Nakatsuji, H.; Caricato, M.; Li, X.; Hratchian, H.P.; Izmaylov, A.F.; Bloino, J.; Zheng, G.; Sonnenberg, J.L.; Hada, M.; Ehara, M.; Toyota, K.; Fukuda, R.; Hasegawa, J.; Ishida, M.; Nakajima, T.; Honda, Y.; Kitao, O.; Nakai, H.; Vreven, T.; Montgomery, Jr., J.A.; Peralta, J.E.; Ogliaro, F.; Bearpark, M.; Heyd, J. J.; Brothers, E.; Kudin, K.N.; Staroverov, V.N.; Kobayashi, R.; Normand, J.; Raghavachari, K.; Rendell, A.; Burant, J.C.; Iyengar, S.S.; Tomasi, J.; Cossi, M.; Rega, N.; Millam, J.M.; Klene, M.; Knox, J.E.; Cross, J.B.; Bakken, V.; Adamo, C.; Jaramillo, J.; Gomperts, R.; Stratmann, R.E.; Yazyev, O.; Austin, A.J.; Cammi, R.; Pomelli, C.; Ochterski, J.W.; Martin, R.L.; Morokuma, K.; Zakrzewski, V.G.; Voth, G.A.; Salvador, P.; Dannenberg, J.J.; Dapprich, S.; Daniels, A.D.; Farkas, Ö.; Foresman, J.B.; Ortiz, J.V.; Cioslowski, J.; Fox, D.J. Gaussian 09, Rev. C 01; Gaussian, Inc., Wallingford CT, **2009**.

[3] Stephens, P. J.; Harada, N. ECD cotton effect approximated by the Gaussian curve and other methods. Chirality **2010**, *22*, 229–233.
